# Bispyrene Functionalization Drives Self-Assembly of Graphite Nanoplates into Highly Efficient Heat Spreader Foils

**DOI:** 10.1021/acsami.1c00319

**Published:** 2021-03-25

**Authors:** Giuseppe Ferraro, M. Mar Bernal, Fabio Carniato, Chiara Novara, Mauro Tortello, Silvia Ronchetti, Fabrizio Giorgis, Alberto Fina

**Affiliations:** † Dipartimento di Scienza Applicata e Tecnologia, Politecnico di Torino, Alessandria Campus, Viale Teresa Michel 5, Alessandria 15121, Italy; ‡ Dipartimento di Scienze e Innovazione Tecnologica, Università degli Studi del Piemonte Orientale “Amedeo Avogadro”, Viale Teresa Michel, 11, Alessandria 15121, Italy; § Dipartimento di Scienza Applicata e Tecnologia, Politecnico di Torino, C.so Duca degli Abruzzi 24, Torino 10129, Italy

**Keywords:** molecular junctions, graphene-related materials, thermally conductive nanopapers, π-gelators, heat spreader, supramolecular functionalization, graphite nanoplates self-assembly

## Abstract

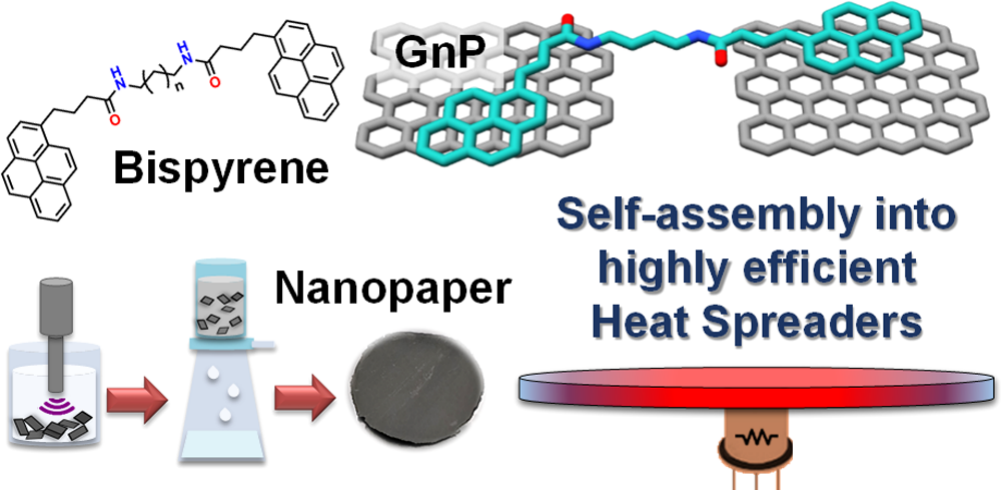

Thermally conductive nanopapers fabricated from graphene and related materials are currently showing great potential in thermal management applications. However, thermal contacts between conductive plates represent the bottleneck for thermal conductivity of nanopapers prepared in the absence of a high temperature step for graphitization. In this work, the problem of ineffective thermal contacts is addressed by the use of bifunctional polyaromatic molecules designed to drive self-assembly of graphite nanoplates (GnP) and establish thermal bridges between them. To preserve the high conductivity associated to a defect-free sp^2^ structure, non-covalent functionalization with bispyrene compounds, synthesized on purpose with variable tethering chain length, was exploited. Pyrene terminal groups granted for a strong π–π interaction with graphene surface, as demonstrated by UV–Vis, fluorescence, and Raman spectroscopies. Bispyrene molecular junctions between GnP were found to control GnP organization and orientation within the nanopaper, delivering significant enhancement in both in-plane and cross-plane thermal diffusivities. Finally, nanopapers were validated as heat spreader devices for electronic components, evidencing comparable or better thermal dissipation performance than conventional Cu foil, while delivering over 90% weight reduction.

## Introduction

Thermal management in modern electronic devices requires the development of new generation of materials to guarantee for both flexibility and heat dissipation, in particular for applications in flexible electronics^1^ as well as in wearable and implantable devices.^2,3^ Graphene was demonstrated as a good candidate for heat management, based on its outstanding thermal conductivity^4−6^ and mechanical properties.^7^ Although single layer graphene currently remains of limited availability for applications in bulk materials, a wide family of graphene related materials (GRM) has become largely available, including reduced graphene oxide (RGO), multilayer graphene (MLG), and graphite nanoplates (GnP).^8^ GRM were indeed largely reported in the literature for the preparation of thermally conductive polymer nanocomposites^9,10^ and nanopapers.^11^ Freestanding GRM nanopapers appear as promising alternatives to the metal foils traditionally used as heat spreaders in a number of heat management applications, coupling high thermal conductivity with flexibility, low density, and chemical stability in harsh environmental conditions. Nanopapers are conveniently produced by vacuum filtration of GRM suspensions through a porous membrane filter,^12−16^ exploiting their self-assembly onto the membrane upon the removal of solvent. Being a facile and scalable process, this approach was largely explored with GO suspended in water.^17−19^ However, owing to the extensive disruption of sp^2^ carbon lattice upon graphene oxidation,^20^ GO membranes exhibit low thermal conductivity values and therefore require proper reduction and thermal annealing to recover sufficient thermal conductivity. Thermal reduction of GO membranes at variable temperature demonstrated thermal conductivities to increase with annealing temperatures, yielding values up to 1000 Wm^–1^K^–1^ for annealing above 1000 °C^21^ and even higher values for annealing above 2000 °C,^11^ when extensive graphitization of the film occurs. Preparation of conductive nanopapers from low-oxidized GRM was also explored and proven effective for thermal conductivity.^22−27^ This latter route allows avoiding the harsh chemical processes for GO production and subsequent reduction of the film, but may lead to lower thermal conductivity (often in the range of 300 Wm^–1^K^–1^) compared to high-temperature annealed GO papers, despite the high thermal conductivity of the individual low-defectiveness nanoplates. This is mainly related to the thermal resistance associated to weak contacts between conductive particles, explained by the weak interaction forces between nanoplates, as well as their limited flexibility and planarity. In the absence of a high temperature and pressure treatment of nanopapers, porous and low-density nanopapers are typically obtained.^11^ While the presence of air pockets is an obvious reason depressing the thermal conductivity of the film, the limited contact area between the conductive nanoplates and the poor thermal conductance across nanoplates contacts are further negatively affecting the nanopapers thermal conductivity. Enhancing the area and quality of contacts between nanoplates is therefore a must for the improvement of nanopapers thermal conductivity. Although higher density, orientation, and contact area may be obtained by compression,^28^ the enhancement of contact quality is significantly more challenging. Molecular junctions have been explored both theroretically^29−32^ and experimentally^33,34^ to produce thermal bridges between conductive particles, enhancing the phonon transfer across the contacts. Effectiveness of molecular junctions for thermal conductance improvement was found to depend on both length and structure of the molecule.^29,30^ However, obtaining precise functionalization of GRM with covalently bound molecular junctions, without disrupting the carbon sp^2^ structure, currently remains very challenging. As an alternative approach for the preparation of thermal bridges between GRMs, supramolecular functionalization based on π–π stacking^35,36^ may also be explored by the use of multifunctional molecules, potentially able to cross-link graphene sheets, without the introduction of structural defects.

**Figure 1:**
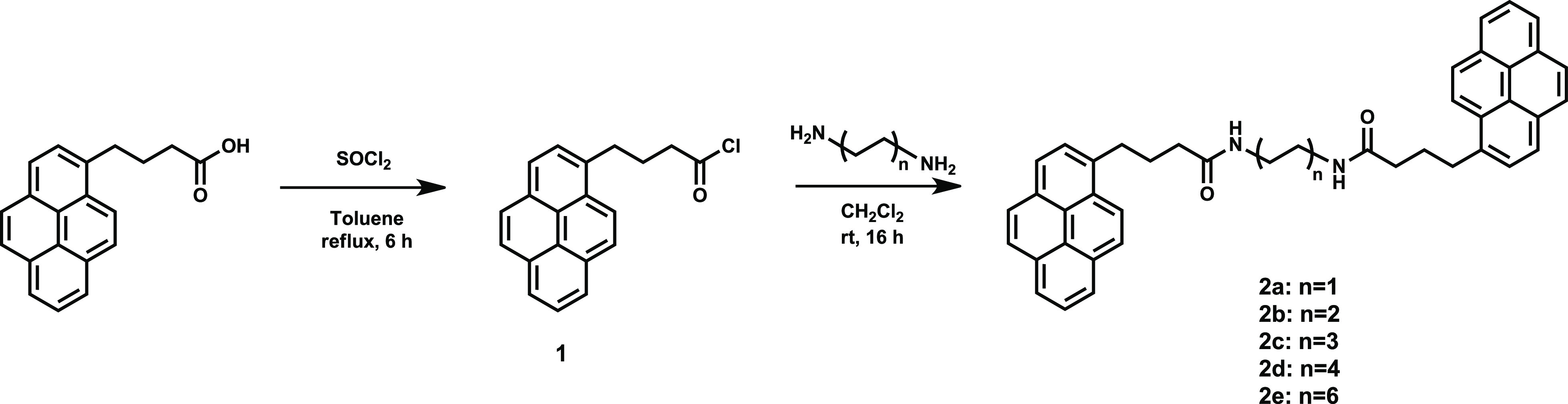
Synthesis of bispyrene molecules 2a–2e.

In this work, bispyrene (BP) molecules (Figure 1) with variable tethering chain length were designed, synthesized, and exploited for the supramolecular functionalization of GnP, to drive their self-assembly and to enhance thermal conductance between thermally conductive nanoplates. Results demonstrated significant enhancement in both in-plane and cross-plane thermal diffusivities, depending on the length of the BP molecule. Most importantly, short BP-functionalized nanopapers were found to provide better heat dissipation efficiency than conventional copper foil, while delivering over 90% weight reduction.

## Results and Discussion

Aiming at the non-covalent cross-linking of GnP, bifunctional molecules able to provide a sufficiently strong surface interaction with graphene layers were designed and synthesized. Among the different chemical species able to strongly interact with the sp^2^ carbon surface via π–π stacking, pyrene was selected based on its well-known adsorption on the graphene surface and its good solubility in organic solvents. A series of bispyrene (BP) molecules (referred to as 2a–2e) were synthesized (Figure 1), varying the length of the alkyl chain connecting the pyrene units. Routine characterization by UV–Vis, NMR, and mass spectrometry is reported in the Supporting Information, Section S1.

**Figure 2:**
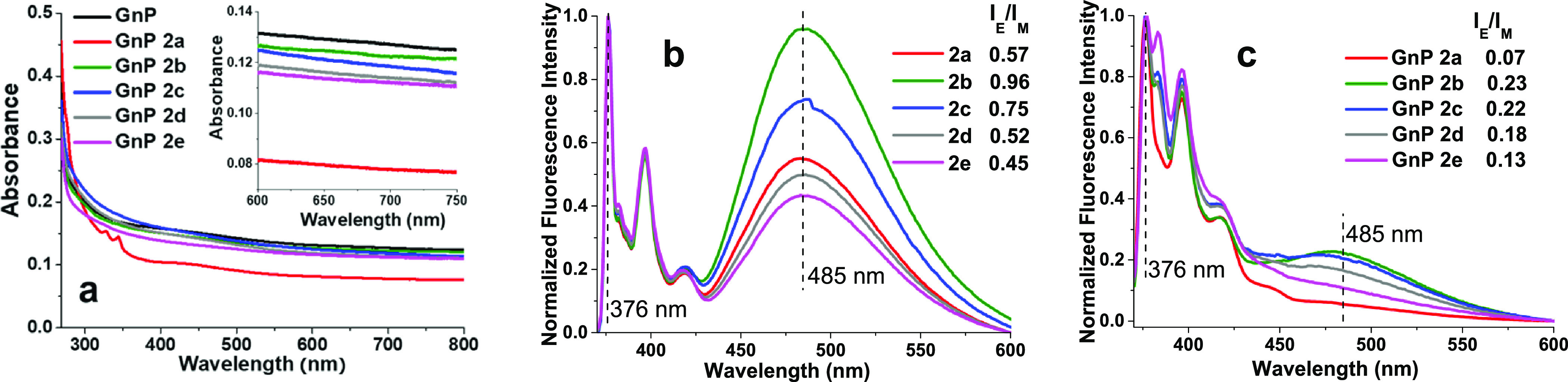
UV–Vis spectra of supernatants (a) inset showing a magnification for the range 600 to 750 nm; fluorescence spectra for BP solutions in DMF, 10^–6^ M (b); and fluorescence spectra for supernatant of GnP/BP dispersions (c).

The interaction of BP molecules with GnP in the suspension was investigated as a function of the alkyl chain length between the pyrene units, by the analysis of supernatants obtained after mild sonication of GnP in solutions of BPs. Initially, the effect of BP concentrations was explored by UV–Vis and fluorescence spectroscopy to verify absorption and emission of these compounds on the GnP surface, while avoiding large excess of free molecules in the systems. Based on the results obtained (Supporting Information, Section S2), a BP concentration of 10^–6^ M was selected to prevent extensive self-aggregation of BP molecules on the surface of the nanoflakes. By UV–Vis absorbance (Figure 2a), a rough estimation of the concentration of GnP suspended in the supernatant can be obtained by analyzing the light absorption at 670 nm wavelength at which BPs do not absorb (spectra in the Supporting Information, Section S1.6). The concentration of suspended GnP in the presence of BP molecules is similar or lower to those of pristine GnP, suggesting that the presence of BPs does not stabilize GnP in suspension but rather promotes aggregation of GnP, acting as π–π gelators, as previously reported for similar systems.^37^ The spectrum for GnP 2a supernatant, in which reduction in the GnP concentration is remarkable, clearly shows the characteristic absorption bands of pyrene groups in the 300–450 nm range,^38^ completely absent in the spectra of supernatants containing longer BP. These facts suggest 2a to be the most efficient BP in promoting the aggregation of GnP, at the given concentration. This result might be interpreted as a consequence of the lower tethering chain length on the molecular conformations for BP interacting with the GnP surface. Fluorescence emission spectra of BP in DMF solution (10^–6^ M, Figure 2b) show distinctive features for both monomer (M, at 376, 396, and 418 nm) and excimer (E, at 485 nm) emissions, with a variable I_E_/I_M_ intensity ratio (Figure 2b), suggesting the highest intramolecular aggregation for BP with intermediate chain lengths (2b and 2c). Fluorescence emission spectra for GnP BP supernatants (Figure 2c) show a strong reduction of I_E_/I_M_ for all BPs, which is consistent with a strong BP/GnP interaction and consequent reduction in the concentration of free BP.

**Figure 3:**
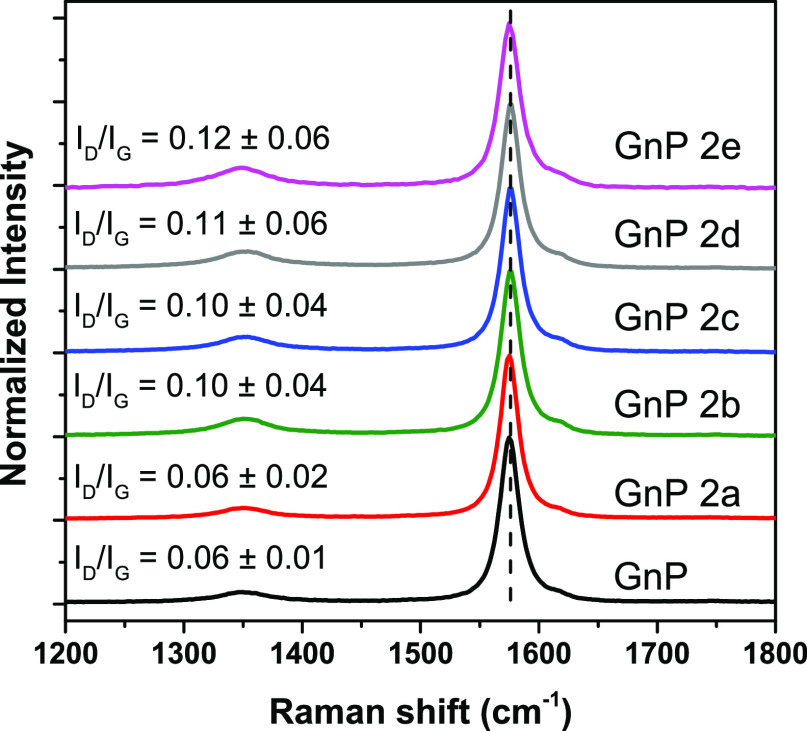
Raman spectra of GnP and BP- functionalized GnP. I_D_/I_G_ band ratio are reported for each of the GnP.

The collected GnP/BP precipitates were thoroughly washed and dried to obtain a series of supramolecular functionalized GnP. Raman spectroscopy was carried out to investigate vibrational changes, which may be correlated to the absorption of BP. In particular, the D band (approx. 1340 cm^–1^), activated by graphene structural defects by second-order Raman scattering processes, the G band (around 1580 cm^–1^) due to first-order Raman scattering (degenerate E_2g_ mode), and in particular, the ratio between their intensities (I_D_/I_G_) can be taken as a parameter for quantifying structural disorder.^39,40^ The low I_D_/I_G_ ratio (0.06) of pristine GnP evidences for a low defectiveness of these nanoflakes (Figure 3). Limited or no increase in average I_D_/I_G_ values were found for BP-functionalized GnP (Figure 3), which is consistent with the non-covalent interaction of pyrene onto the GnP basal plane, not expected to significantly affect its vibrational properties.

Based on the above evidence for strong adsorption of BP and promoted interactions between GnP flakes, nanopapers were fabricated by gravimetric filtration of GnP-BP suspensions, with the aim of exploiting bispyrenes to drive self-assembly between GnP flakes. The amount of BP retained within the nanopapers after filtration and washing was calculated from the concentration of BP infiltrated solutions, compared to the initial concentration in the GnP/BP suspension. The retention rate of BP into the nanopapers (Table 1) was found to be ≥95%, except in the case of GnP 2a (80%, reflecting the higher concentration of 2a in the supernatant). The mass fraction of BP in the nanopapers is ranging between 0.09% for 2a and 0.15% for 2e, which increase may be partially explained by the higher molar mass of longer chain BP.

**Table 1:** Extent of Functionalization and Physical Properties for GnP and BP-GnP Nanopapers

nanopaper	BP retention rate [%]	BP mass fraction [%]	density [g cm^–3^]	in-plane thermal diffusivity [mm^2^ s^–1^]	cross-plane thermal diffusivity [mm^2^ s^–1^]
GnP			1.22 ± 0.05	175 ± 11	0.4 ± 0.1
GnP 2a	80 ± 1	0.09 ± 0.01	0.62 ± 0.02	204 ± 10	2.2 ± 0.2
GnP 2b	95 ± 1	0.12 ± 0.01	0.67 ± 0.02	192 ± 10	2.3 ± 0.2
GnP 2c	95 ± 1	0.13 ± 0.01	0.75 ± 0.02	168 ± 13	1.5 ± 0.3
GnP 2d	99 ± 1	0.13 ± 0.01	0.77 ± 0.02	167 ± 15	1.3 ± 0.7
GnP 2e	99 ± 1	0.15 ± 0.01	0.94 ± 0.02	172 ± 10	0.6 ± 0.2

Such low mass fractions depend on the limited surface area of GnP (38 m^2^/g, for the as-received powder), as well as the low concentration of BP. These values correspond to a very limited coverage of the GnP with BP moieties, in the range of μmol BP for gram of GnP, corresponding to a number of BP molecules per GnP surface in the order of 10^4^/μm^2^ (assuming retained specific surface area upon GnP suspension in DMF) or lower. Such low coverage was designed to provide sufficient GnP functionalization while avoiding self-aggregation of BP into the nanopapers, which is indeed possible at higher concentrations, as demonstrated by FESEM analysis (Supporting Information, Section S3). Densities of nanopapers were found to be significantly different from the reference GnP nanopaper (1.22 g cm^–3^). In fact, lower densities were obtained for all of the GnP-bispyrene nanopapers, ranging between 0.62 and 0.94 g cm^–3^. Interestingly, density values continuously increase with increasing the length of the BP alkyl chain in the GnP-bispyrene nanopaper (Table 1). This trend is not straightforwardly explained in terms of BP mass fraction. Instead, it appears that the density of the GnP-bispyrene nanopaper depends on the interactions between BPs and GnP, as proven by the characterization of suspensions, driving the self-assembling of GnP during filtration.

**Figure 4:**
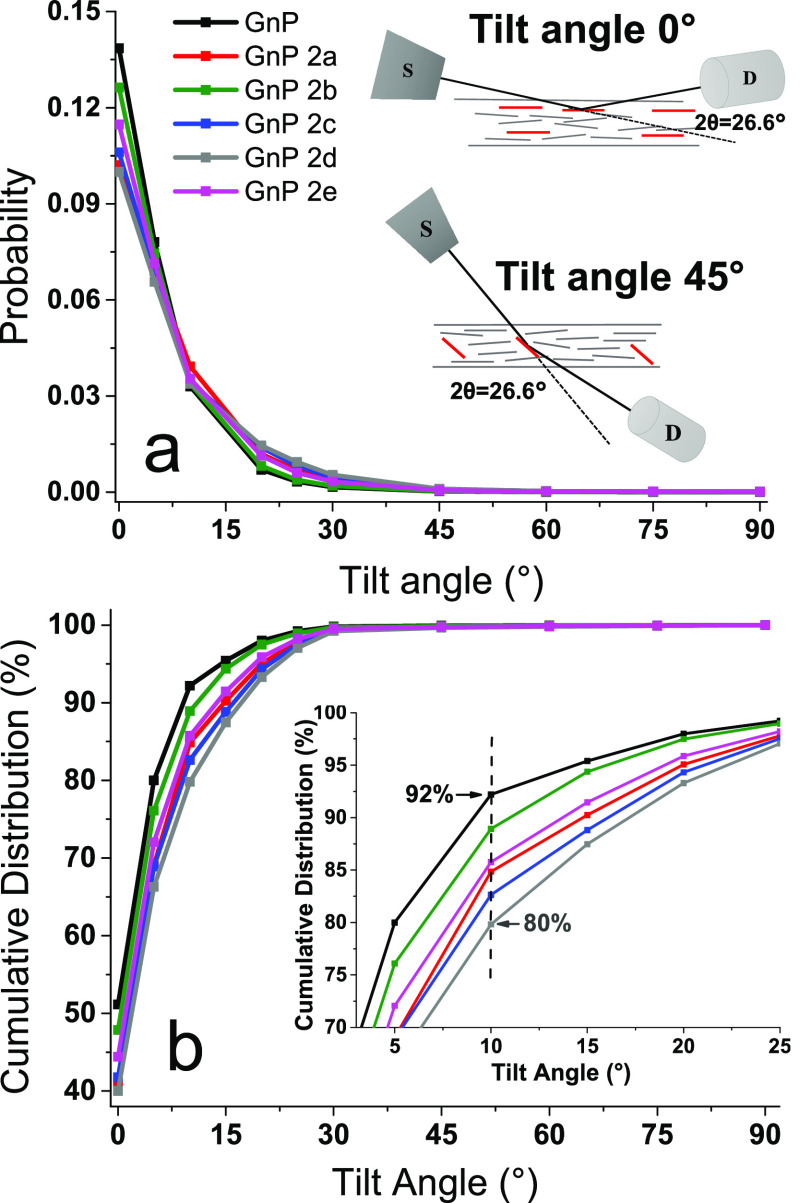
(a) Probability distribution for the orientation of GnP flakes, based on the 002 diffraction signal for basal planes in graphite. Inset shows a schematic representation of configuration used, as a function of incident angle while maintaining constant 2θ = 26.6°. In red are highlighted GnP flakes contributing to the diffracted beam, oriented at 0 and 45° tilt angles, respectively. S is for the X-ray source, D is for the X-ray detector. (b) Cumulative distribution for GnP orientation *vs* tilt angle. Inset shows a magnification in the tilt angle range 3 to 25°.

To further investigate the assembly of nanopapers, X-ray diffraction (XRD) was exploited to evaluate the orientation of flakes into the films, monitoring the intensity of the graphite (002) diffraction peak at 26.6° 2θ, as a function of X-ray beam incident angle. Intensities were normalized to obtain a distribution of probability for nanoflakes orientation (details in the Supporting Information, Section S4), as a function of tilt angle, from 0° (002 basal planes parallel to the nanopaper plane) to 90° (perpendicular to the plane). Although the filtration of GnP suspensions is obviously expected to produce a clear in-plane orientation of GnP flakes, comparing the distribution of the basal planes orientations for functionalized *vs* pristine GnP nanopapers may indeed provide insight in the assembly of GnP. The orientation for 002 planes in GnP (Figure 4a) is clearly maximum in the direction parallel to the nanopaper (0° tilt angle) for all nanopapers. However, the maximum probability values and the intensity decay profile with the tilt angle are different for GnP and the GnP-BP nanopapers. The cumulative distributions (Figure 4b) provide a quantification of preferential orientation. For pristine GnP, approx. 92% of the cumulative distribution of flakes has an orientation between 0 and 10° tilt angle (i.e., an arrangement almost parallel to the surface of the nanopaper), whereas for GnP-bispyrene nanopaper in the same tilt angle range, cumulative distribution vary approx. in the range between 80 and 88%. Despite no clear trend can be drawn as a function of the tethering chain length in BP, these values evidence for a slightly lower in-plane orientation of the GnP-bispyrene nanopaper, compared to pristine GnP, confirming BPs to have a role in the self-assembly of GnP during filtration. This result is consistent with the lower densities observed for functionalized GnP nanopapers and is likely explained by BP-bridged aggregation of suspended GnP flakes into clusters, which in turn constraints the organization of flakes during filtration, decreasing the packing factor, and eventually reducing preferential orientation and density.

**Figure 5:**
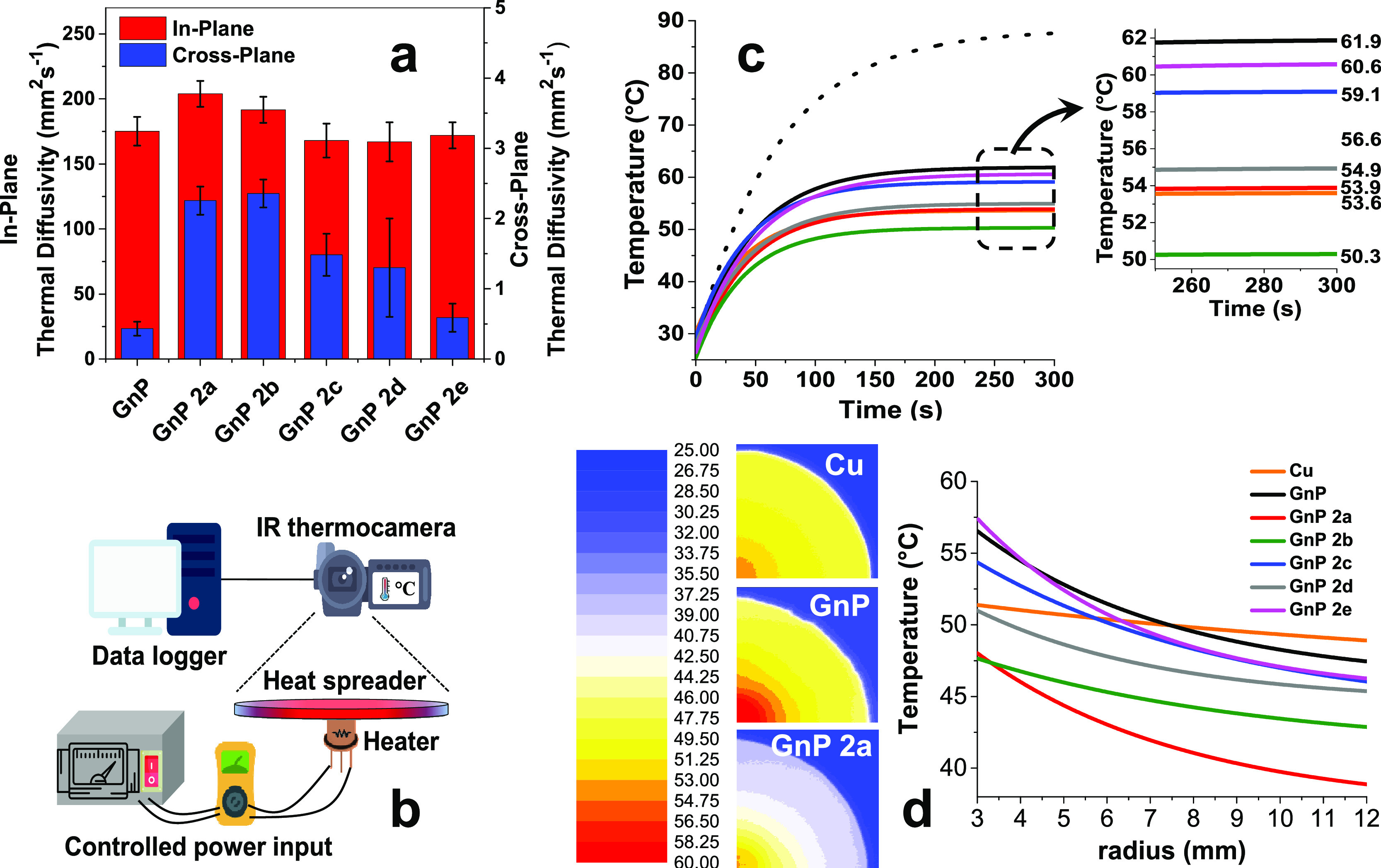
Thermal properties of GnP nanopapers. (a) In-plane and cross-plane thermal diffusivities for GnP and GnP-bispyrene nanopapers; (b) setup for the measurement of thermal properties as heat spreader; (c) hotspot temperature profile *vs* time for GnP nanopapers *vs* copper foil; (d) thermal imaging at 300 s of selected heat spreaders (I quadrant only for better visibility) and fitted temperature gradient *vs* radius comparison for GnP nanopapers *vs* copper foil (details are reported in the Supporting Information, S7).

Insight on the heat spreading efficiency of nanopapers was obtained by measuring thermal diffusivity, i.e. the rate of heat transfer, within the porous material. Both in-plane and cross-plane diffusivities were measured for pristine GnP and GnP-bispyrene nanopapers (Table 1 and Figure 5a), evidencing for significant differences induced by BP functionalization. Thermal conductivity values for different nanopapers, calculated from the diffusivity values, density, and heat capacity, are discussed in the Supporting Information, Section S5. As expected from the orientation of GnP, thermal diffusivity in nanopapers is strongly anisotropic, with at least two orders of magnitude higher diffusivity in plane, compared to cross-plane. Cross-plane thermal diffusivity for GnP/BP nanopapers are typically higher than those of pristine GnP, which may be partially related to the above described lower nanoflakes orientation. Indeed, the presence of even very limited portions of flakes lying on directions tilted with respect to the nanopaper plane is expected to contribute to the heat transfer across the nanopaper, owing to the well-known anisotropy of graphitic materials. However, the changes in cross-plane diffusivity do not match the misalignment degree, as evaluated by XRD, suggesting a different explanation about the role of BP moieties. Short BPs (2a and 2b) resulted to be the most effective functionalization for the in-plane diffusivity enhancement, suggesting these molecules to reduce the thermal resistance between overlapped nanoflakes. This may be explained by promoted aggregation between GnP, maximizing their contact area, as well as by the enhancement of phonon transfer efficiency through BP molecules bridging adjacent GnP, thus acting as non-covalent molecular junctions for phonon transfer. BP 2a and 2b also significantly enhanced in-plane thermal diffusivity, whereas values for GnP-bispyrene nanopapers containing longer BPs are equivalent to pristine GnP. The in-plane diffusivity enhancement is obtained in spite of the lower in-plane orientation for GnP-bispyrene nanopaper, thus further supporting the role of BP molecular junctions in the reduction of thermal resistance at GnP-GnP contacts. It is also worth mentioning that the described trend in thermal diffusivity is not corresponding to significant changes in volumetric electrical conductivity for the nanopapers, measured in the range of 2 × 10^5^ S m^−1^ (Supporting Information, Section S6), highlighting that the effect of BP molecules is specifically on thermal transport properties.

From an application perspective, such an increased in-plane thermal diffusivity may be exploited in heat spreader applications, used for the dissipation of heat from a hotspot, over a larger surface. This typically occurs in several electronic components used in modern devices, especially in high frequency processors, where the excess heat must be efficiently dissipated to prevent potential failures. Copper is traditionally used for heat spreaders, in the form of foils or finned structures. GnP nanopapers are in principle a valid alternative to metals, owing to their flexibility and low density, thus opening for applications in flexible and lightweight devices, such as flexible electronics as well as wearable and implantable devices. With the aim of comparing performances of GnP-bispyrene nanopapers with respect to the conventional metal foil, a simple experimental setup (Figure 5b) for the measurement of heat spread from a hotspot was built. Using a powered transistor, simulating a hotspot and an IR thermocamera to collect thermal maps in time, the temperature evolution over the heat spreader, made with the copper film or with the GnP nanopapers, was continuously monitored during heating (power on, Figure 5c) and cooling (power off, Figure S30) of the hotspot. In the absence of a heat spreader foil, the temperature of the hotspot raises to about 85 °C after 300 s power on. As expected, the presence of copper foil strongly reduced the hotspot temperature to 53.6 °C at 300 s (Figure 5c, inset), as the heat is distributed on the surface of the foil and eventually dissipated to the surrounding air, by natural convection. Copper foil was used as a benchmark, based on its well-known thermal conductivity in the range of 400 Wm^–1^K^–1^. With nanopaper heat spreaders, temperature profiles *vs* time were found strongly dependent on the presence and type of functionalization (Figure 5c). Indeed, while all GnP-bispyrene nanopaper have a maximum hotspot temperature lower than those with the pristine GnP, nanopaper GnP 2a revealed to be as effective as Cu foil and GnP 2b demonstrated even better performance, allowing to maintain a hotspot temperature (50.3 °C at 300 s) constantly lower than in the presence of copper foil. This outstanding result is even more impressive when considering the densities of the heat spreaders, as the porous GnP 2b nanopaper is only 0.67 g cm^–3^, whereas copper is about 8.9 g cm^–3^, thus delivering a straightforward weight reduction of the heat spreader by one order of magnitude.

To further investigate the heat dissipation over the spreader foils, the thermal maps acquired in time, upon heating and cooling the transistor, were analyzed to calculate the thermal gradient on the foil *vs* the radial coordinate (Supporting Information, Section S7). Selected temperature maps reported in Figure 5d clearly evidence for significant differences in the temperature gradient over the surface for copper, GnP, and BP-GnP. In particular, a lower temperature gradient is apparent on Cu foil, compared to GnP nanopapers, evidencing for an efficient distribution of heat over the surface, according to its high thermal conductivity value. A comparison between temperature *vs* radius profiles for Cu and GnP nanopapers (Figure 5d and Figures S31–S33) confirms a systematically higher temperature decay rate on the GnP foils, suggesting a lower capability to distribute the heat flow over the nanopapers, likely related to their lower density compared to copper. These results may appear contradictory to the thermal diffusivity results, but it has to be recalled that the heat flux is dependent on thermal conductivity, which in turn depends linearly on the density of the dissipating materials. Given the relatively low bulk density of GnP and the porosity of the nanopapers (see Supporting Information, Section S5 for a detailed discussion), the heat spreading capability is indeed expected to be lower than that for a high-density metal. Therefore, the demonstrated performance of GnP nanopapers to keep the hotspot at low temperature appears to be related to phenomena beyond the bare heat conduction. In particular, the overall performance of the heat spreader may also strongly depend on thermal transfer at the solid/air interface. While emissivity of the surface is leveled off by the use of black paint on all the heat spreader, surface properties associated to the porosity, and surface roughness of GnP nanopapers appear to control heat dissipation, eventually leading to a performance comparable to or even higher than copper foil.

## Conclusions

Graphite nanoplates (GnP) were functionalized with a new family of bispyrene (BP) molecules, designed to anchor pyrene moieties on GnP and connect them via an aliphatic chain with variable length. Synthesized BP demonstrated strong adsorption onto GnP and tendency to promote aggregation of nanoflakes in suspension, thus suggesting potential in the self-assembly of GnP. Functional GnP foils were manufactured via the gravimetric filtration method and referred to as nanopapers, owing to similarities with the well-known paper-making process.

GnP organization within nanopapers was evaluated by electron microscopy and X-ray diffraction, to quantify porosity and orientation of the flakes within the nanopapers, demonstrating a strong role of BP in the self-assembly of GnP, leading to lower densities and reduced preferential in-plane orientation. Thermal diffusivity of GnP nanopapers was found to depend on the length of BP, with shortest BPs delivering best thermal diffusivity in both cross-plane and in-plane diffusivities. Indeed, while GnP nanopaper showed 175 mm^2^ s^–1^ in-plane and 0.4 mm^2^ s^–1^ cross-plane thermal diffusivity, GnP 2a nanopapers exhibited thermal diffusivity values of 204 mm^2^ s^–1^ and 2.2 mm^2^ s^–1^ for in-plane and cross-plane diffusivity, respectively. These evidences support for promoted aggregation between GnP, maximizing their contact area and enhancing phonon transfer efficiency within the network of conductive flakes, demonstrating bispyrene molecules as effective drivers for a thermally efficient self-assembly of GnP.

To demonstrate the performance of GnP nanopapers in conditions representative of actual heat spread applications, a simple experimental setup was used to simulate cooling of an overheated electronic device (as a representative hotspot). In these conditions, GnP nanopapers were benchmarked to a copper foil with the same geometry. Hotspot temperature measures demonstrated that all BP-functionalized nanopapers perform better than pristine GnP nanopapers. Most importantly, one functionalized nanopaper demonstrated better performance in cooling the hotspot, compared to the copper benchmark. Beside thermal performance, a dramatic 90% weight reduction was obtained for the nanopapers, compared to copper foil, which may open for potential application of thermally conductive nanopapers in a number of lightweight and flexible devices.

## Materials and Methods

Graphite nanoplates (GnP) with a mean size of some tens of micrometers and a thickness of few nanometers were prepared and provided by Avanzare Innovación Tecnólogica S.L (Navarrete, La Rioja, Spain) via rapid thermal expansion of overoxidized-intercalated graphite, according to a previously reported procedure.^41^ Detailed characterization of this GnP was previously reported.^10,41^

### Synthesis of Bispyrene Molecules

1-pyrenebutyric acid (300 mg, 1.4 mmol) (Sigma Aldrich, 97%) was dissolved in 100 mL of dry toluene (Sigma Aldrich, anhydrous, 99.8%) and cooled at 5 °C. Thionyl chloride (Sigma Aldrich, ≥99.0%) (10 mL) was added dropwise, and the mixture was heated to reflux for 6 h under an argon atmosphere. The solvent was evaporated in vacuum, obtaining an orange solid, which was subsequently dissolved in dichloromethane (20 mL) (Sigma Aldrich, anhydrous, ≥99.8%). A solution of the diaminoalkane (0.5 mmol, 1,2-diaminoethane, Sigma Aldrich, ≥99.5%; 1,4-diaminobutane, Sigma Aldrich, ≥99%; 1,6-diaminohexane, Sigma Aldrich, ≥99%; 1,8-diaminooctane, Sigma Aldrich, 98%; 1,12-diaminododecane, Sigma Aldrich, 98%) in dichloromethane (2 mL) was added dropwise to the solution containing 1-pyrenebutyric acid, and the reaction mixture was stirred at room temperature for 18 h. The precipitate was filtered using nylon filter membranes (pore size 0.45 μm, Whatman), washed with dichloromethane, and the solvent was evaporated. To remove the non-reacted diaminoalkane, the solid was stirred for 1 h in a 10^–4^ M solution of hydrochloric acid (Sigma Aldrich, 37%) and filtered to obtain the final bispyrene (BP) product. Nuclear magnetic resonance analysis (NMR) was acquired on Bruker AVANCE III (Italy) at 500 MHz (∼3 mg of powder were solubilized into 1 mL of hexadeuterodimethyl sulfoxide, Sigma Aldrich, 99.96 atom % D). Liquid chromatography coupled with mass spectrometry (LC–MS) was performed to acquire the spectra (samples <1 mg solubilized into 5 mL of methanol for HPLC, ≥99%) with an electrospray ionization H^+^ mode (model LTQ XL, Thermo Fisher Scientific, USA).

### Functionalization of GnP with BP

GnP (10 mg) were added to a solution (20 mL of the selected BP in *N*,*N*-dimethylformamide (10^–6^ M) (DMF, Sigma Aldrich, anhydrous, 99.8%) at a concentration of 0.5 mg mL^–1^. The solutions were sonicated in pulsed mode (5 s on/5 s off) for 30 min at a power output of 150 W by using an ultrasonication probe (Sonics Vibracell VCX-750, Sonics & Materials Inc., USA) with a 5 mm diameter Ti-alloy tip. After that, the suspension was left to decant for 120 min at 5 °C and part of the supernatant was carefully collected for further characterization, while the other part was filtrated through a polytetrafluoroethylene (PTFE) supported membrane (0.2 μm as pore size, Whatman), washed with deionized water (50 mL), ethanol (50 mL, Carlo Erba, 96%), and diethyl ether (50 mL, Sigma Aldrich, anhydrous, ≥99.7%), and dried at 60 °C for 24 h. Raman spectra were acquired on a Renishaw inVia Reflex (Renishaw PLC, UK) Raman microscope using an excitation laser wavelength of 514.5 nm. The measurements were performed using a 20× objective in backscattering configuration, setting the laser power to 2.5 mW and the integration time to 50 s. The reported spectra are the average of five measurements acquired in different areas of the sample. Peaks have been fitted with a Lorentzian function using the origin Pro 2020 software. The supernatant was centrifuged at 4000 rpm for 30 min and left to decant overnight. Finally, the part above the sediment (supernatant) was sampled and analyzed by UV–Visible spectroscopy, (UV-2600, Shimadzu, Japan) with a single scan of a 0.5 nm sampling interval and 0.05 s accumulation time, 1 cm quartz cuvette, and the photoluminescence spectra were recorded on a Horiba Jobin-Yvon model IBH FL-322 Fluorolog 3 spectrometer equipped with a 450 W xenon arc lamp with excitation at 345.00 nm.

### Preparation and Characterization of GnP-BP Nanopapers

GnP and BP-functionalized GnP were suspended on *N*,*N*-dimethylformamide (Sigma Aldrich, anhydrous, 99.8%) solutions (0.5 mg mL^–1^) and sonicated in pulsed mode (5 s on and 5 s off) for 30 min with a power set at 150 W) by using an ultrasonication probe (Sonics Vibracell VCX-750, Sonics & Materials Inc., USA) with a 13 mm diameter Ti-alloy tip. The suspensions were gravimetrically filtrated using a polyamide membrane (0.45 μm as pore size, Whatman) and then fixed in a petri glass with Scotch tape to be dried at 65 °C under vacuum for 2 h to completely remove the solvent. Filtrated solutions were collected and analyzed by UV–Vis (UV-2600, Shimadzu, 1 cm quartz cuvette with a single scan of a 0.5 nm sampling interval, and 0.05 s accumulation time) to measure the BP absorbance and calculate the concentration from relative calibration line using *y* = *m*·*x* equation (where *y* is the absorbance, *m* is the slope of the curve, and *x* is the concentration of the solution). Mass fraction of BP adsorbed onto GnP was calculated from the difference between initial concentration and concentration in the filtered solution.

Nanopapers were peeled off from the membranes and were mechanically pressed in a laboratory hydraulic press (Atlas 15 T, Specac, England) under a uniaxial compressive load of 5 kN for 30 min at 25 °C. Nanopapers were die-cut into 0.53 mm diameter disks to calculate the densities, as the ratio between mass measured using a microbalance (resolution 1 μg, TA Discovery TGA used at room temperature) and the volume calculated from the known diameter and thickness, measured by field-emission scanning electron microscopy (FESEM, Zeiss Merlin 4248, beam voltage: 5 kV, Germany) on the cross section of the GnP nanopapers. Thicknesses of the nanopapers were typically measured in the range between 20 and 40 μm (see cross-sectional micrographs in Figure S24).

Orientations of GnP in the nanopapers were evaluated using an X-ray Panalytical X’Pert PRO (Cu Kα radiation) diffractometer, with a PIXcel detector, a solid-state detector with rapid readout time and high dynamic range. The specimens (disks 23 mm diameter,) were supported onto the PET film (5 μm) and measured with a 2θ scan axis for each value of the incident angle (α) ranging from 13 to 103° with respect to the horizontal plane of the sample. Intensities of the signal of 002 graphite planes (2θ = 26.6°) were collected against the incident angle. Intensities are reported as a function of the tilt angle α-θ_002_, which corresponds to the tilt angle of the flakes in the nanopaper, varying from ∼0° (basal planes parallel to the nanopaper plane) to ∼90° (basal planes perpendicular to the nanopaper plane). The intensity *vs* 2θ experimental curves (for each incident angle) were fitted with a Lorentzian function using the OriginPro 2020 software to obtain the peak intensity for the 002 diffraction signal. Then, the peak intensities *vs* tilt angle were plotted for each of the nanopapers and fitted by an exponential decay curve using OriginPro 2020 software. Finally, peak intensities *vs* tilt angle plots were normalized on their integral value, to obtain the probability distribution for the flakes oriented from parallel to perpendicular to the nanopaper plane.

The in-plane thermal diffusivity (α_∥_) and cross-plane diffusivity (α_⊥_) were measured at 25 °C by the xenon light flash analysis (LFA) (Netzsch LFA 467 Hyperflash*,* Germany). The samples were die-cut in disks of 23 mm, and the measurement of the α_∥_ was carried out in an in-plane sample holder, while the α_⊥_ was measured in the standard cross-plane configuration. At least three different specimens were tested for each of the nanopaper formulations. Five measurements were collected on each sample to calculate average values and experimental deviations.

### Heat Spreader Device Setup

Heat spread performance was evaluated using the same specimen geometry used for LFA, assembled onto a transistor (2N2222 A, low power bipolar transistor, NPN silicon planar switching transistor, TO-18 metal package, STMicroelectronics), powered by an electrical generator (GWinstec GPS-3303, Taiwan), set at 0.080 A current at 4.3 V to provide 0.345 W. The thermal contact of a nanopaper disk heat spreader onto the flat surface (12.56 mm^2^) of the transistor is mechanically guaranteed by a 3 × 3× 3 mm^3^ NdFeB (N28) magnet, centered onto the transistor top surface. The heat spreader disks (35 μm of copper foil or GnP nanopapers with comparable thickness), as well as the magnetic cube were black paint sprayed to control their emissivity. The temperature of the system was monitored using an IR thermal imaging camera (Optris-Cam PI-400, Germany), with an optical resolution of 382 × 288 pixel and 1 Hz sampling rate. Tests were carried out under a closed box measuring 29 × 23 × 19 cm^3^ to ensure the reproducibility of natural convection upon heating (power on) for 300 s and cooling (power off) for another 300 s. Thermal maps on the magnet surface were elaborated using OriginPro 2020 software. Hotspot temperature was calculated as the average temperature over the 3 × 3 mm^2^ area in top of the magnet, which is directly related to the temperature of the transistor. Temperature profiles *vs* radial coordinate along three different directions (considering a Cartesian *x*, *y* system with the origin centered in the center of the sample, the horizontal, vertical, and oblique at 45° directions of the first quadrant) were extracted and averaged to obtain a representative temperature *vs* radius decay curve.

## Supporting Information Available

Additional characterization of bispyrene molecules, GnP functionalization, and nanopapers structure and properties (PDF)
