# Supplementary material for: Bispyrene Functionalization Drives Self-Assembly of Graphite Nanoplates into Highly Efficient Heat Spreader Foils
Source: ACS Appl Mater Interfaces. 2021 Mar 25;13(13):15509–17. doi: 10.1021/acsami.1c00319 (PMC8033565; doi:10.1021/acsami.1c00319)
Supplement: Supplementary file 1 — am1c00319_si_001.pdf [file am1c00319_si_001.pdf]

Supporting Information

Bispyrene functionalization drives self-assembly of graphite nanoplates into highly efficient heat spreader foils

Giuseppe Ferraro[1], M. Mar Bernal[1], Fabio Carniato[2], Chiara Novara[3], Mauro Tortello[3], Silvia Ronchetti[3], Fabrizio Giorgis[3], Alberto Fina[1,]*

Dipartimento di Scienza Applicata e Tecnologia, Politecnico di Torino, Alessandria Campus, Viale Teresa Michel 5, 15121 Alessandria, Italy

Dipartimento di Scienze e Innovazione Tecnologica, Università degli Studi del Piemonte Orientale "Amedeo Avogadro", Viale Teresa Michel, 11 - 15121 Alessandria

Dipartimento di Scienza Applicata e Tecnologia, Politecnico di Torino, C.so Duca degli Abruzzi 24, 10129, Torino, Italy

*corresponding author: alberto.fina@polito.it

Index

S1. Characterization of bispyrene molecules 2

S1.1. N,N'-(ethane-1,2-diyl)bis(4-(pyren-1-yl)butanamide) (2a) 2

S1.2. N,N'-(butane-1,4-diyl)bis(4-(pyren-1-yl)butanamide) (2b) 4

S1.3. N,N'-(hexane-1,6-diyl)bis(4-(pyren-1-yl)butanamide) (2c) 6

S1.4. N,N'-(octane-1,8-diyl)bis(4-(pyren-1-yl)butanamide) (2d) 8

S1.5. N,N'-(dodecane-1,12-diyl)bis(4-(pyren-1-yl)butanamide) (2e) 10

S1.6. Comments on UV-Vis spectra 13

S2. Optimization of the concentration of BP for the supramolecular functionalization of GnP with N,N'-(ethane-1,2-diyl)bis(4-(pyren-1-yl)butanamide) (2a) 14

S3. FESEM analysis 19

S4. Nanoflakes orientation via X-ray diffraction 23

S5. Thermal Conductivity of GnP nanopapers 24

S6. Electrical conductivity 27

S7. Heat spreader setup and analysis of temperature profiles 28

S1. Characterization of bispyrene molecules

All reagents and solvents were purchased were purchased from Aldrich, Alfa Aesar, Fisher or Acros Organics and used without further purification unless otherwise stated.

UV-Vis spectra were recorded on a Shimadzu UV-2600, 1 cm quartz cuvette with single scan 0.5 nm sampling interval and 0.05 s accumulation time, in dimethyl formamide.

All ^{1}H and ^{13}C Nuclear Magnetic Resonance (NMR) spectra were recorded on a NMR 500 MHz Bruker AVANCE III. Samples were dissolved in deuterated dimethylsulfoxide (DMSO-d_6) with TMS as internal reference (chemical shifts δ in ppm). The following abbreviations were used to describe spin multiplicity: s = singlet, d =doublet, t = triplet, m = multiplet.

Liquid chromatography coupled with mass spectroscopy (model LTQ XL, Thermo Fisher Scientific, USA) was carried out with Electrospray ionizations (ESI) with <1 mg into 5 ml of methanol (HPLC grade, ≥99%) in which two drops of formic acid (ACS reagent, ≥96%,) have been added to promote protonation.

S1.1. N,N'-(ethane-1,2-diyl)bis(4-(pyren-1-yl)butanamide) (2a)

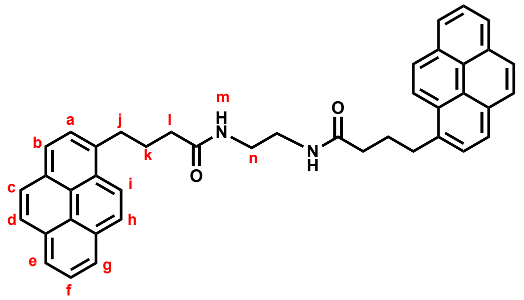

Yield: 262.4 mg (84 %)

UV-Vis absorbance: 344.5, 328.5, 314.5, 277.5, 266.5, 256.5, 244.5, 235.5 nm.

^{1}H NMR (500 MHz, DMSO-d_6, 300 K) δ (ppm): 8.40 (d, J = 9.3 Hz, 1H, g), 8.31 (t, J = 7.2 Hz, 2H, e +d), 8.25 – 8.20 (m, 2H, f+c), 8.17 (s, 2H, b + h), 8.11 (t, J = 7.6 Hz, 1H, m), 7.95 (d, J = 7.7 Hz, 2H, a + i), 3.36 – 3.32 (m, 2H, n), 3.25 – 3.19 (m, 2H, j), 2.29 (t, J = 7.3 Hz, 2H, l), 2.06 (dd, J = 14.9, 7.4 Hz, 2H, k).

^{13}C NMR (126 MHz, DMSO-d_6, 300 K) δ (ppm): 184.02, 172.53, 137.01, 131.34, 130.88, 129.74, 128.60, 127.95, 127.89, 127.63, 126.94, 126.57, 125.35, 125.22, 124.69, 124.61, 123.95, 120.68, 40.52, 40.36, 40.19, 40.02, 39.86, 39.69, 39.52, 39.31, 38.94, 38.13, 37.54, 36.88, 35.53, 32.70, 27.93.

ESI-MS(+): 623.62 $[M+Na]^+$, 331.43 $[M-272$ for 4-(pyren-1-yl)butanal$)+2H]^+$, 311.38 [4-(pyren-1-yl)butanal$+K]^+$.

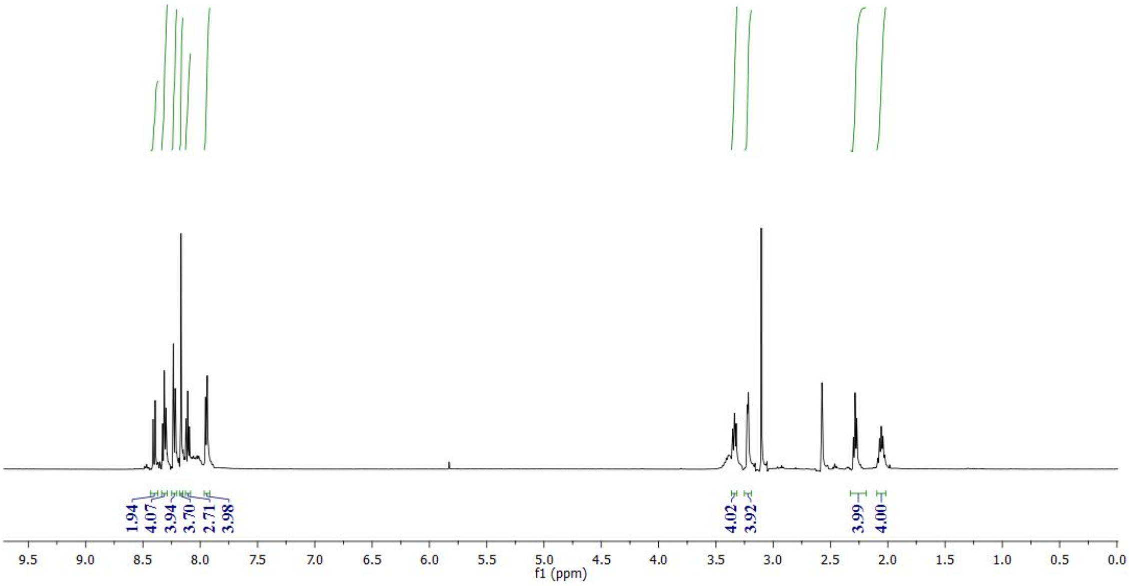

*Figure S1. ^{1}H NMR spectrum of **2a** in DMSO-d_6.*

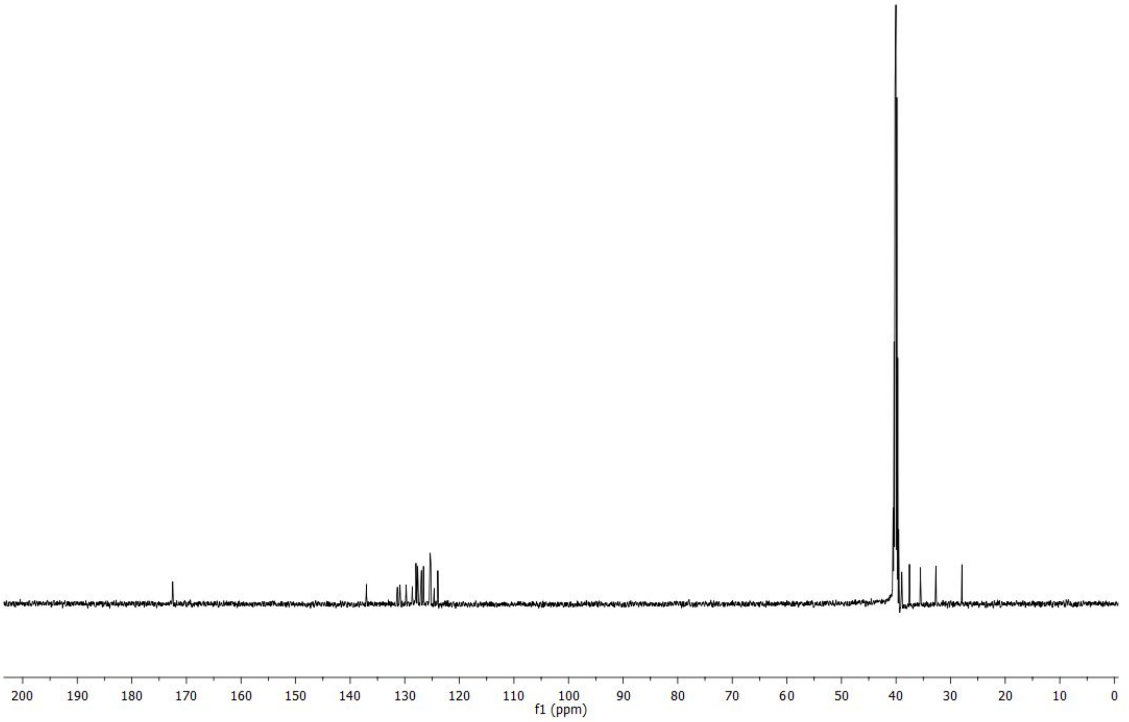

*Figure S2. ^{13}C NMR spectrum of **2a** in DMSO-d_6.*

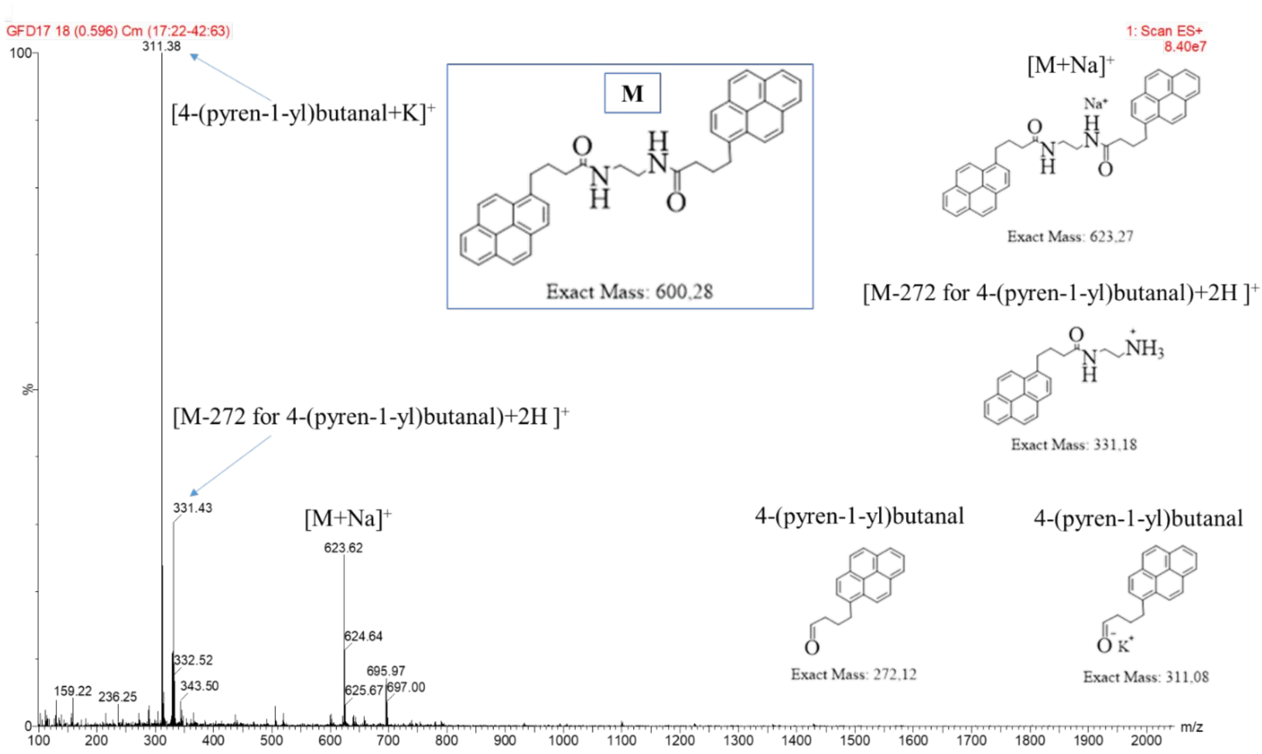

Figure S3: ESI⁺ MS spectrum of ***2a***

S1.2. N,N'-(butane-1,4-diyl)bis(4-(pyren-1-yl)butanamide) (2b)

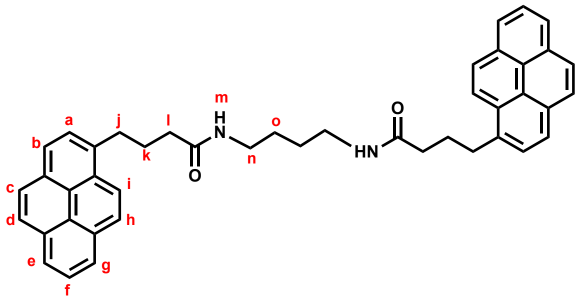

Yield: 260.9 mg (80 %)

UV-Vis absorbance: 344.5, 328.5, 314.5, 277.5, 266.5, 256.5, 244.5, 235.5 nm.

^{1}H NMR (500 MHz, DMSO-d_6, 300 K) δ (ppm): 8.40 (d, J = 9.3 Hz, 1H, g), 8.32 (d, J = 7.6 Hz, 2H, e + d), 8.27 – 8.22 (m, 2H, f + c), 8.17 (s, 2H, b + h), 8.11 (t, J = 7.6 Hz, 1H, m), 7.96 (d, J = 7.8 Hz, 1H, a), 7.88 (t, J = 5.1 Hz, 1H, i), 3.37 – 3.29 (m, 2H, n), 3.14 (d, J = 4.9 Hz, 2H, j), 2.27 (t, J = 7.1 Hz, 2H, l), 2.05 (dt, J = 14.3, 7.2 Hz, 2H, k), 1.48 (s, 2H, o). ^{13}C NMR (126 MHz, DMSO-d_6, 300 K) δ (ppm): 172.14, 127.99, 127.28, 126.94, 126.58, 125.30, 124.64, 123.93, 40.50, 40.34, 39.93, 39.70, 39.59, 38.68, 35.47, 32.71, 28.05, 27.25.

ESI-MS(+): 667.67 [M+K^{+}], 629.69 [M+H]$^{+}$, 359.45 [M-272 for 4-(pyren-1-yl)butanal+2H]$^{+}$.

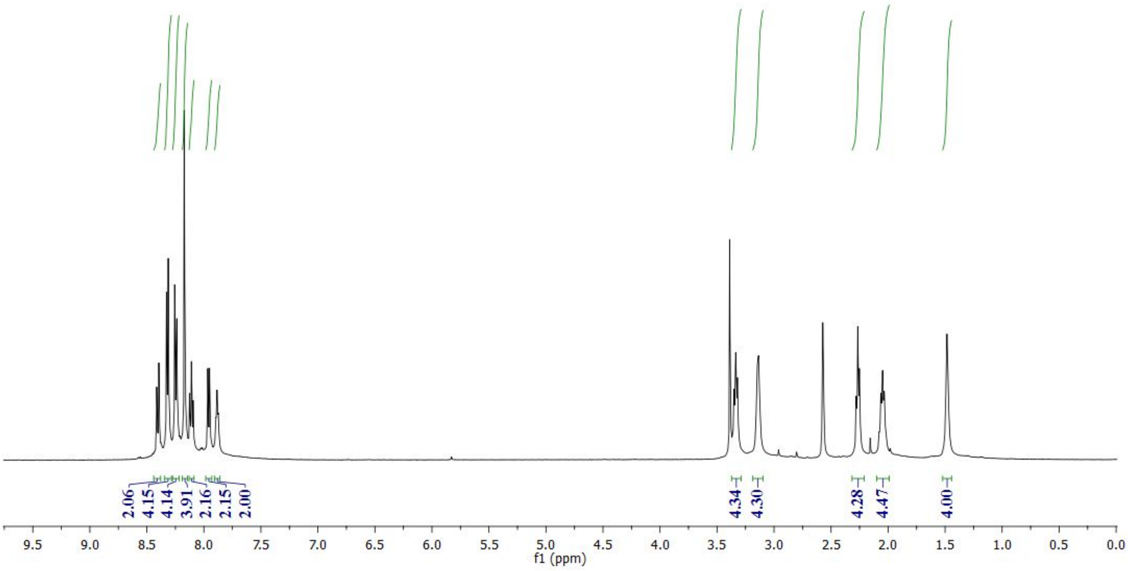

*Figure S4.^{1}H NMR spectrum of **2b** in DMSO-d_6.*

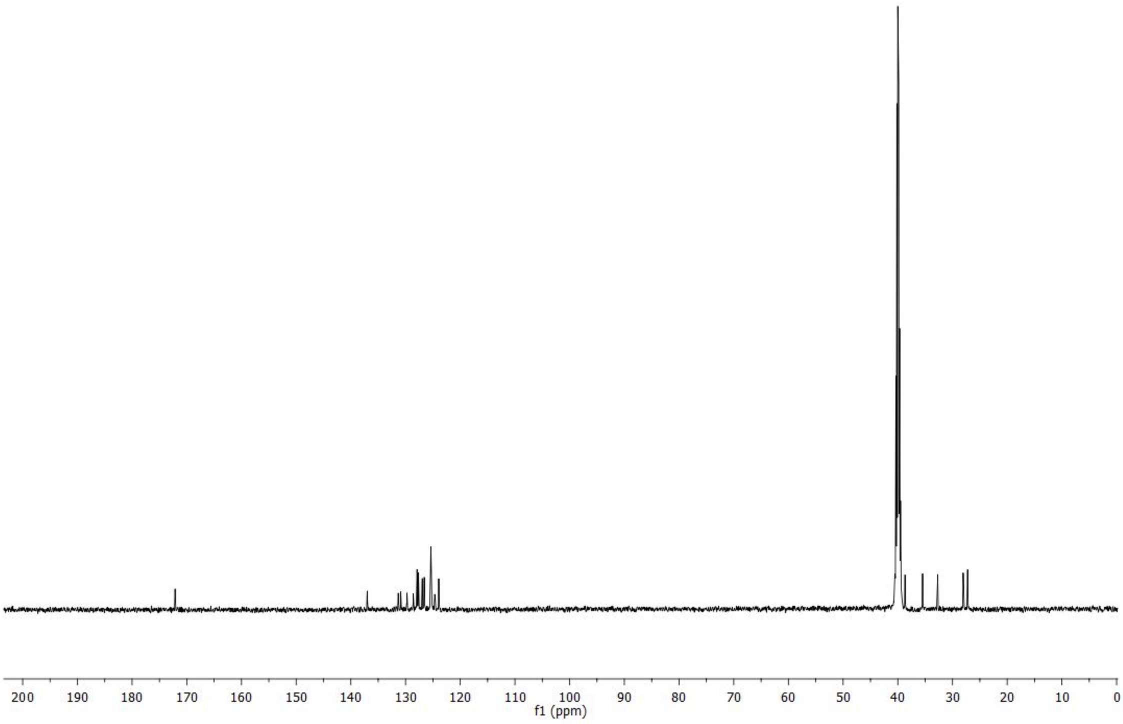

*Figure S5.^{13}C NMR spectrum of **2b** in DMSO-d_6.*

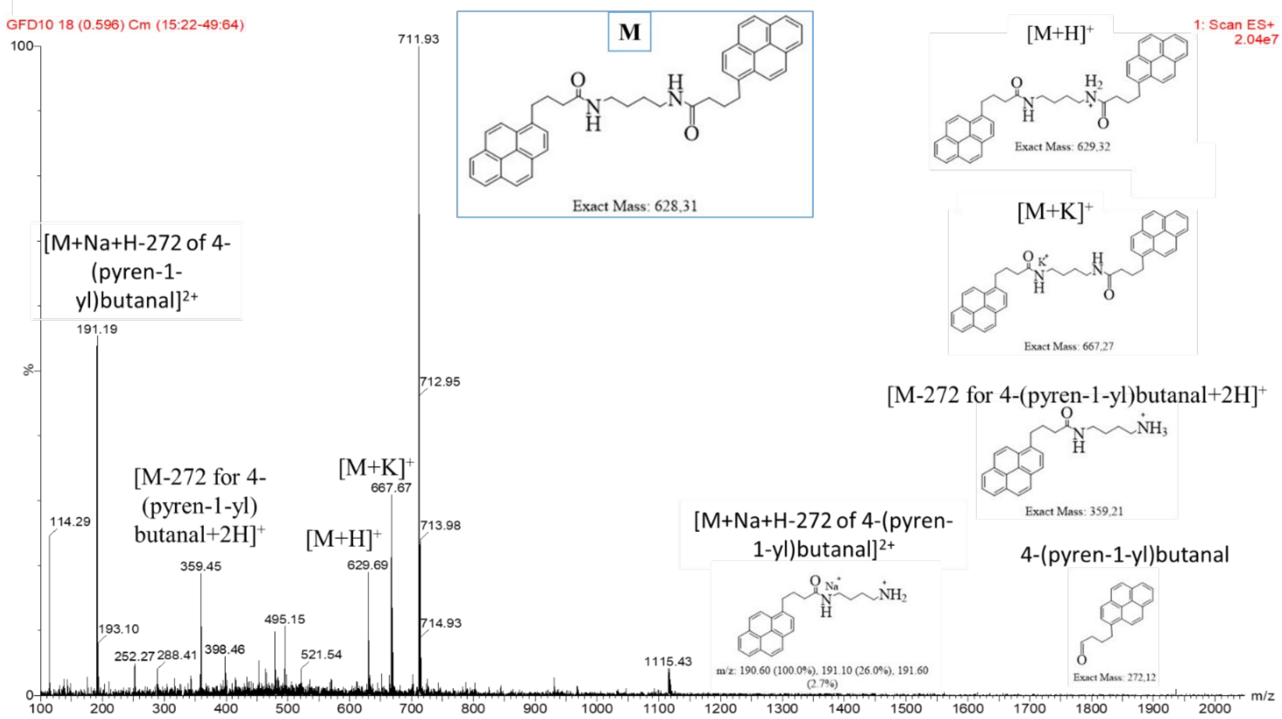

*Figure S6: ESI+ MS spectrum of **2b***

S1.3. N,N'-(hexane-1,6-diyl)bis(4-(pyren-1-yl)butanamide) (2c)

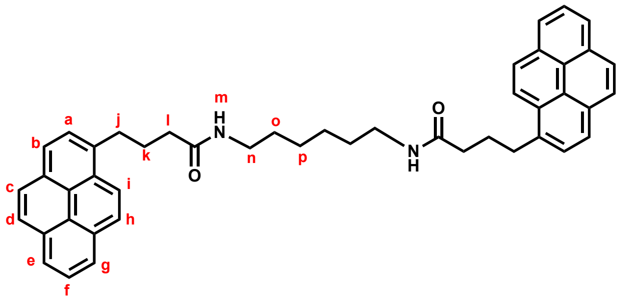

Yield: 262.5 mg (77 %)

UV-Vis absorbance: 344.5, 328.5, 314.5, 277.5, 266.5, 256.5, 244.5, 235.5 nm.

^{1}H NMR (500 MHz, DMSO-d$_6$, 300 K) δ (ppm): 8.42 (d, J = 8.5 Hz, 1H, g), 8.32 (s, 2H, e + d), 8.27 (s, 2H, f + c), 8.24 – 8.14 (m, 2H, b + h), 8.11 (s, 1H, m), 8.04 – 7.92 (m, 1H, a), 7.86 – 7.74 (m, 1H, i), 3.21 – 3.06 (m, 2H, j), 2.37 – 2.21 (m, 2H, l), 2.15 – 2.01 (m, 2H, k), 1.53 – 1.40 (m, 2H, o), 1.41 – 1.28 (m, 2H, p). ^{13}C NMR (126 MHz, DMSO-d$_6$, 300 K) δ (ppm): 172.11, 148.72, 137.03, 131.37, 130.91, 129.77, 128.64, 127.93, 127.90, 127.64, 126.94, 126.56, 125.37, 125.22, 124.73, 124.65, 123.93, 55.35, 38.86, 35.52, 32.69, 29.64, 28.03, 26.62.

ESI-MS(+): 695.91 [M+K]$^+$, 679.74 [M+Na]$^+$.

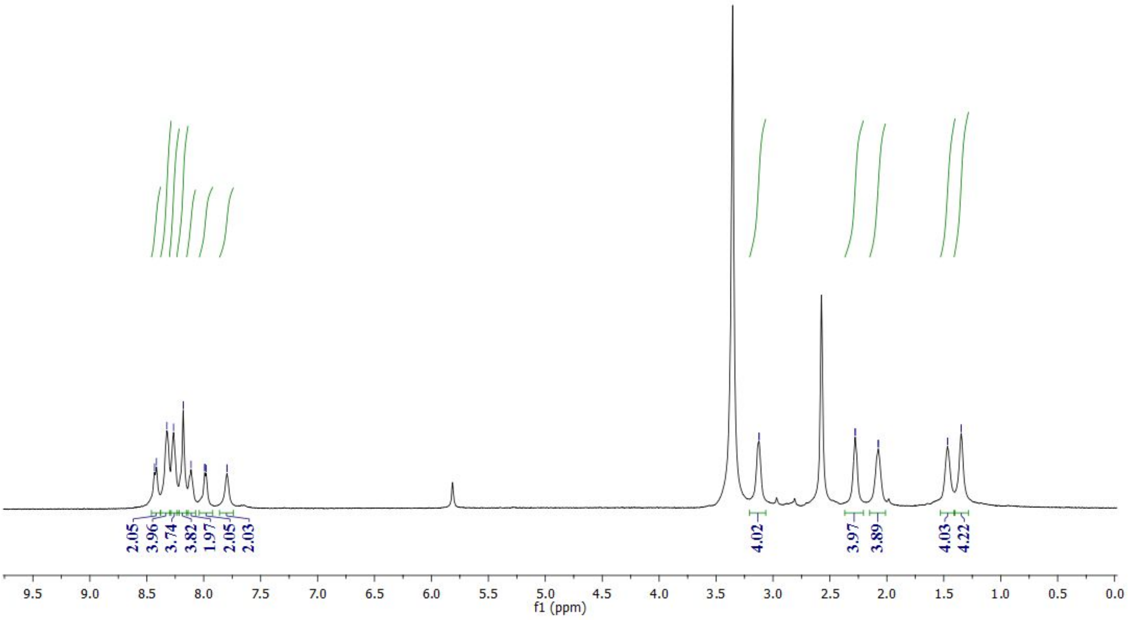

*Figure S7.^{1}H NMR spectrum of **2c** in DMSO-d$_6$.*

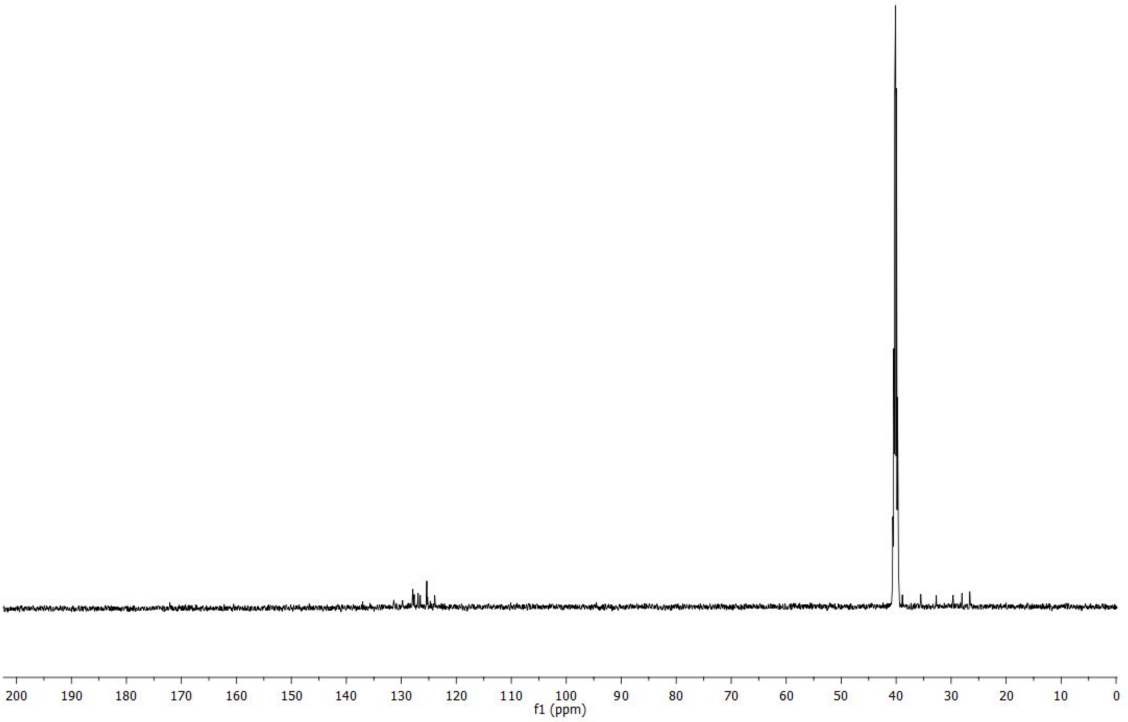

*Figure S8. ^{13}C NMR spectrum of **2c** in DMSO-d$_6$*

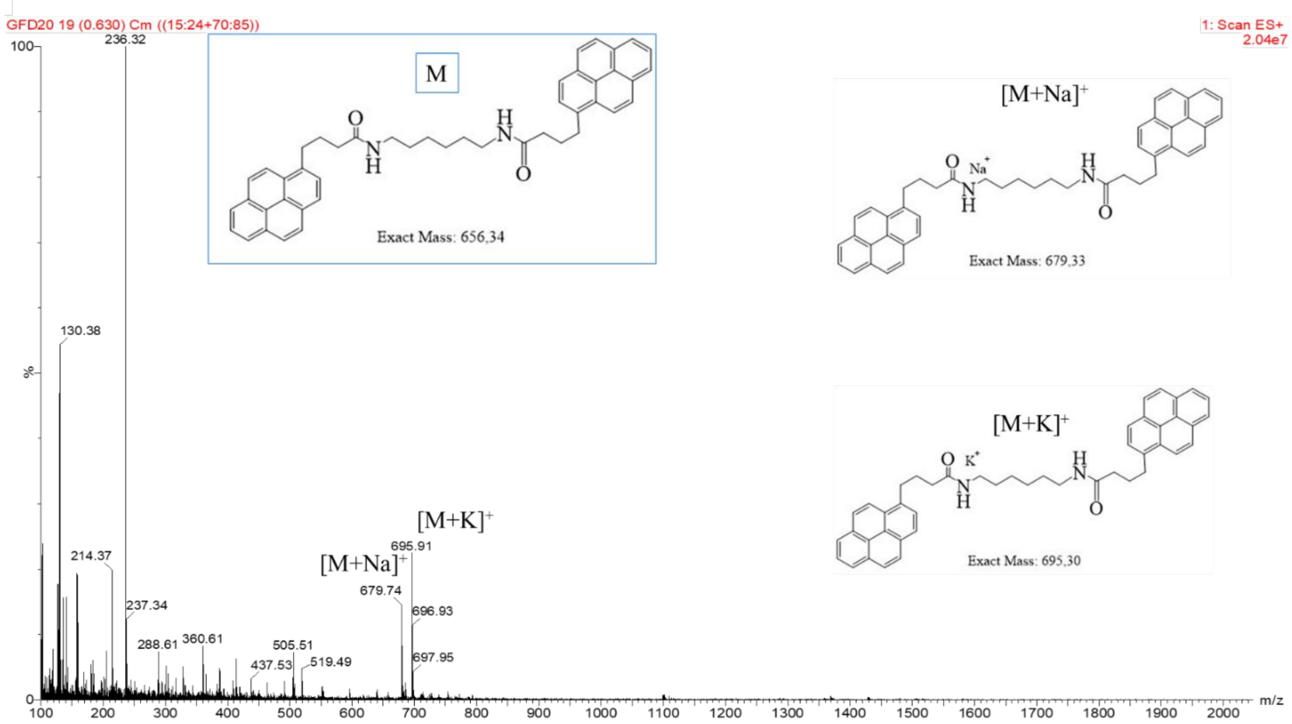

*Figure S9: ESI+ MS spectrum of **2c***

S1.4. N,N'-(octane-1,8-diyl)bis(4-(pyren-1-yl)butanamide) (2d)

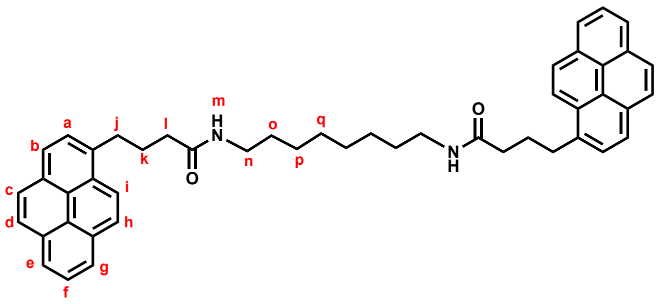

Yield: 255.2 mg (72 %)

UV-Vis absorbance: 344.5, 328.5, 314.5, 277.5, 266.5, 256.5, 244.5, 235.5 nm.

^{1}H NMR (500 MHz, DMSO-d$_6$, 300 K) δ (ppm): 8.47 – 8.40 (m, 1H, g), 8.33 (t, *J* = 7.3 Hz, 2H, e + d), 8.27 (dd, *J* = 8.4, 6.6 Hz, 2H, f + c), 8.18 (q, *J* = 8.9 Hz, 2H, b + h), 8.12 (dd, *J* = 13.7, 6.0 Hz, 1H, m), 8.00 (t, *J* = 8.8 Hz, 1H, a), 7.83 (t, *J* = 5.4 Hz, 1H, i), 3.36 (d, *J* = 7.9 Hz, 2H, n), 3.10 (dd, *J* = 12.7, 6.6 Hz, 2H, j), 2.27 (t, *J* = 7.2 Hz, 2H, l), 2.11 – 2.02 (m, 2H, k), 1.43 (d, *J* = 6.0 Hz, 2H, o), 1.30 (s, 4H p + q). ^{13}C NMR (126 MHz, DMSO-d$_6$, 300 K) δ (ppm): 172.08, 137.03, 127.98, 127.50, 125.31, 40.56, 40.56, 40.04, 40.05, 39.94, 39.60, 32.71, 26.86.

ESI-MS(+): 707.47 [M+Na$^+$], 685.61 [M+H$^+$], 415.43 [M-272 of 4-(pyren-1-yl)butanal+2H]$^+$.

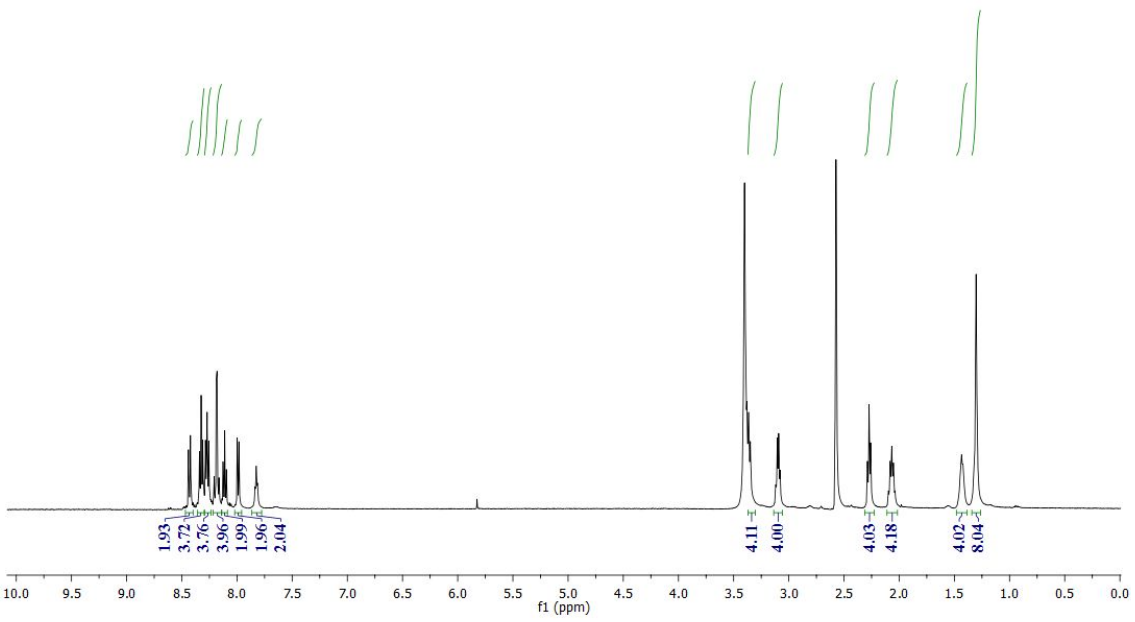

*Figure S10. ^{1}H NMR spectrum of **2d** in DMSO-d_6.*

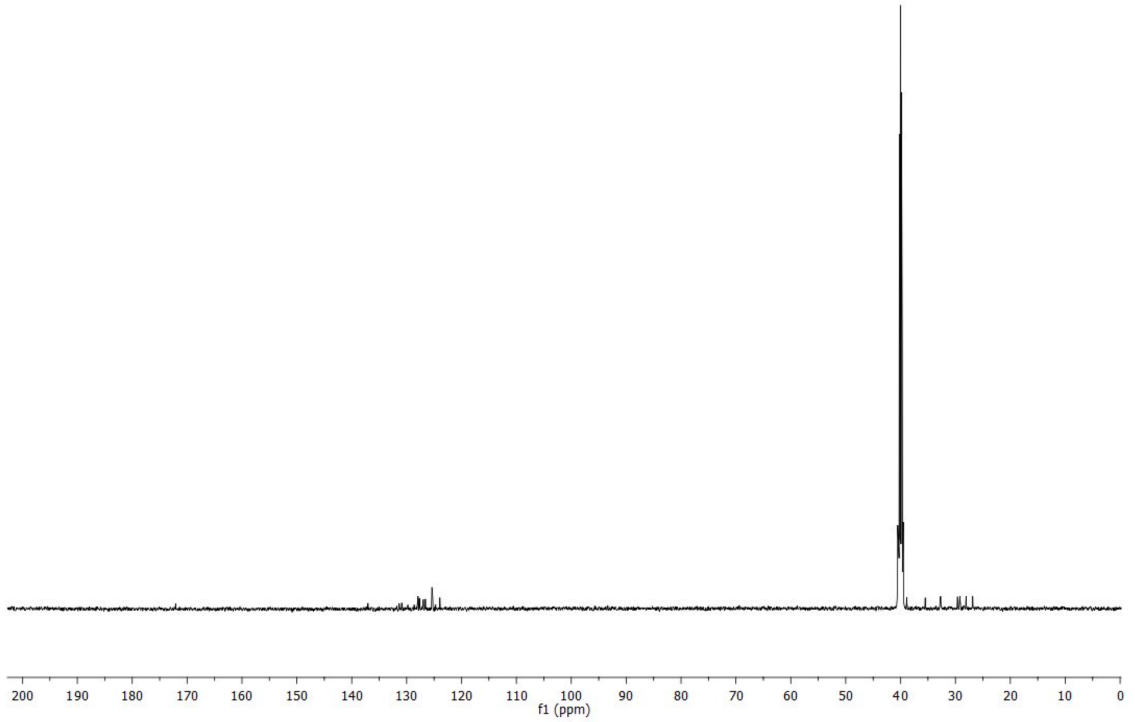

*Figure S11. ^{13}C NMR spectrum of **2d** in DMSO-d_6.*

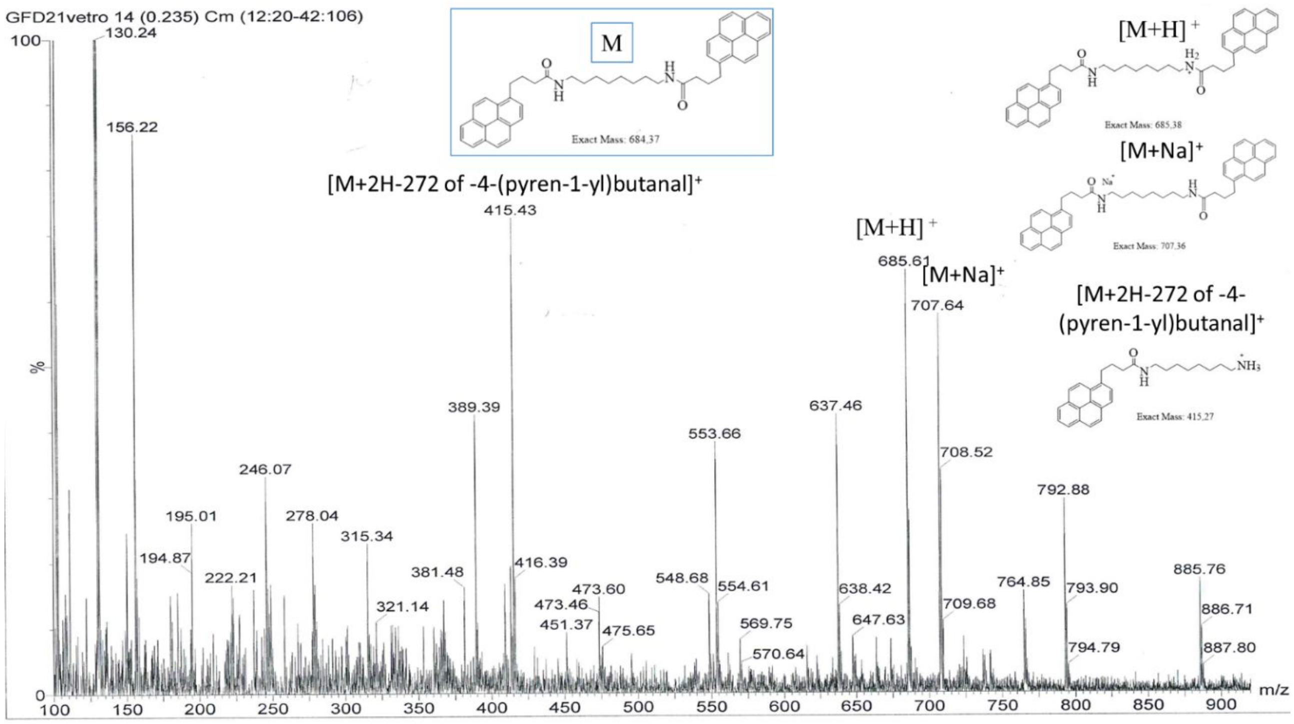

*Figure S12: ESI⁺ MS spectrum of **2d***

S1.5. N,N'-(dodecane-1,12-diyl)bis(4-(pyren-1-yl)butanamide) (2e)

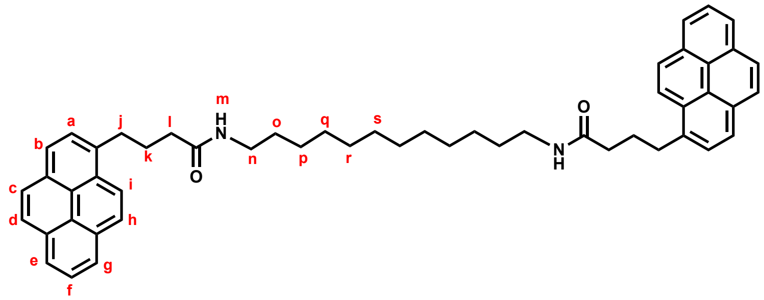

Yield: 246.7 mg (64 %)

UV-Vis absorbance: 344.5, 328.5, 314.5, 277.5, 266.5, 256.5, 244.5, 235.5 nm.

^{1}H NMR (500 MHz, DMSO-d_6, 300 K) δ (ppm): 8.43 (d, J = 9.1 Hz, 1H, g), 8.37 – 8.30 (m, 2H, e + d), 8.31 – 8.23 (m, 2H, f + c), 8.24 – 8.15 (m, 2H, b +h), 8.11 (t, J = 7.7 Hz, 1H, m), 7.99 (d, J = 7.7 Hz, 1H, a), 7.84 (s, 1H, i), 3.36 – 3.32 (m, 2H, n), 3.10 (d, J = 5.9 Hz, 2H, j), 2.28 (d, J = 6.4 Hz, 2H, l), 2.07 (s, 2H, k), 1.41 (s, 2H, o), 1.22 (d, J = 20.6 Hz, 8H, p + q + r + s). ^{13}C NMR (126 MHz, DMSO-d_6, 300 K) δ (ppm): 143.09, 141.40, 127.78, 125.31, 123.94, 40.54, 39.88, 39.95, 39.52, 30.28, 28.54, 28.10, 27.22, 26.13.

ESI-MS(+): 471.49 [M-272 of 4-(pyren-1-yl)butanal+2H]$^+$.

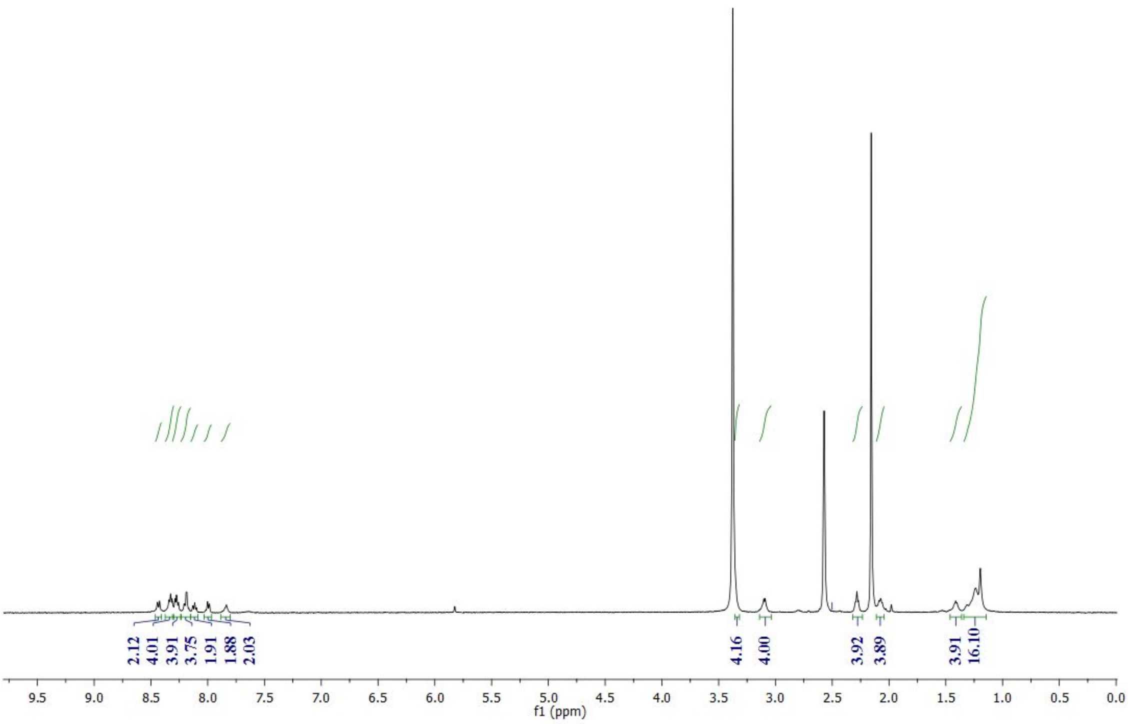

*Figure S13.^{1}H NMR spectrum of **2e** in DMSO-d_6.*

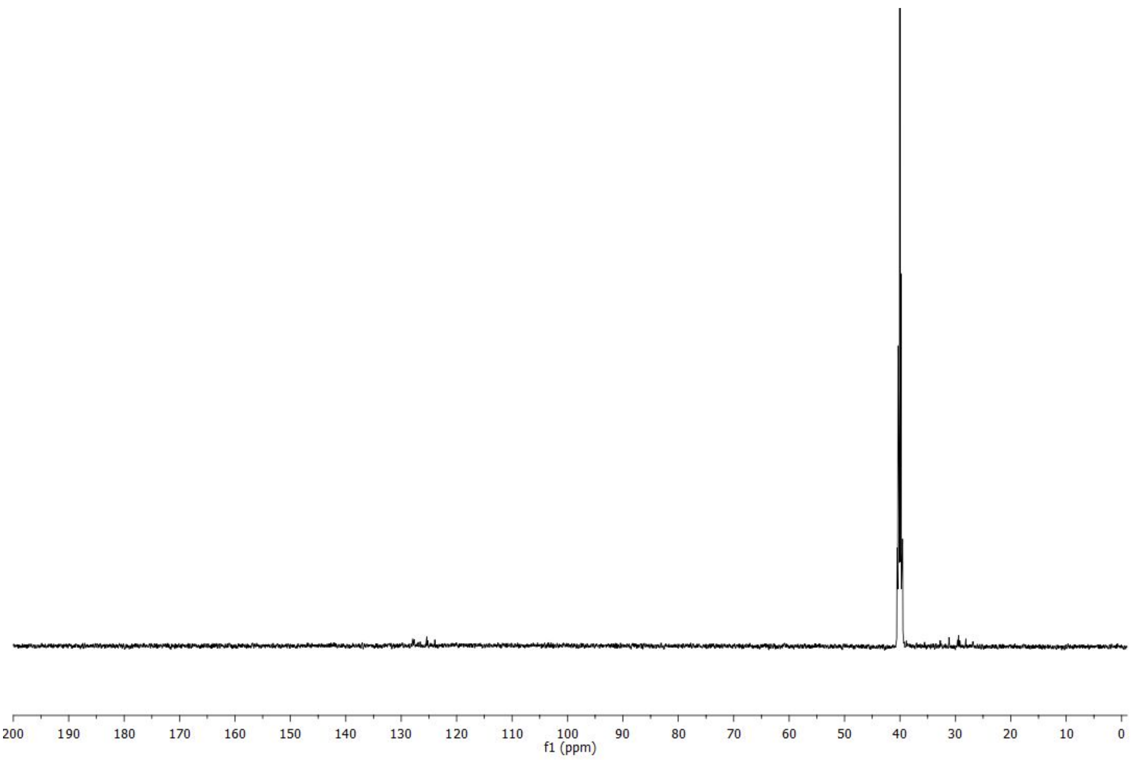

*Figure S14. ^{13}C NMR spectrum of **2e** in DMSO-d_6*

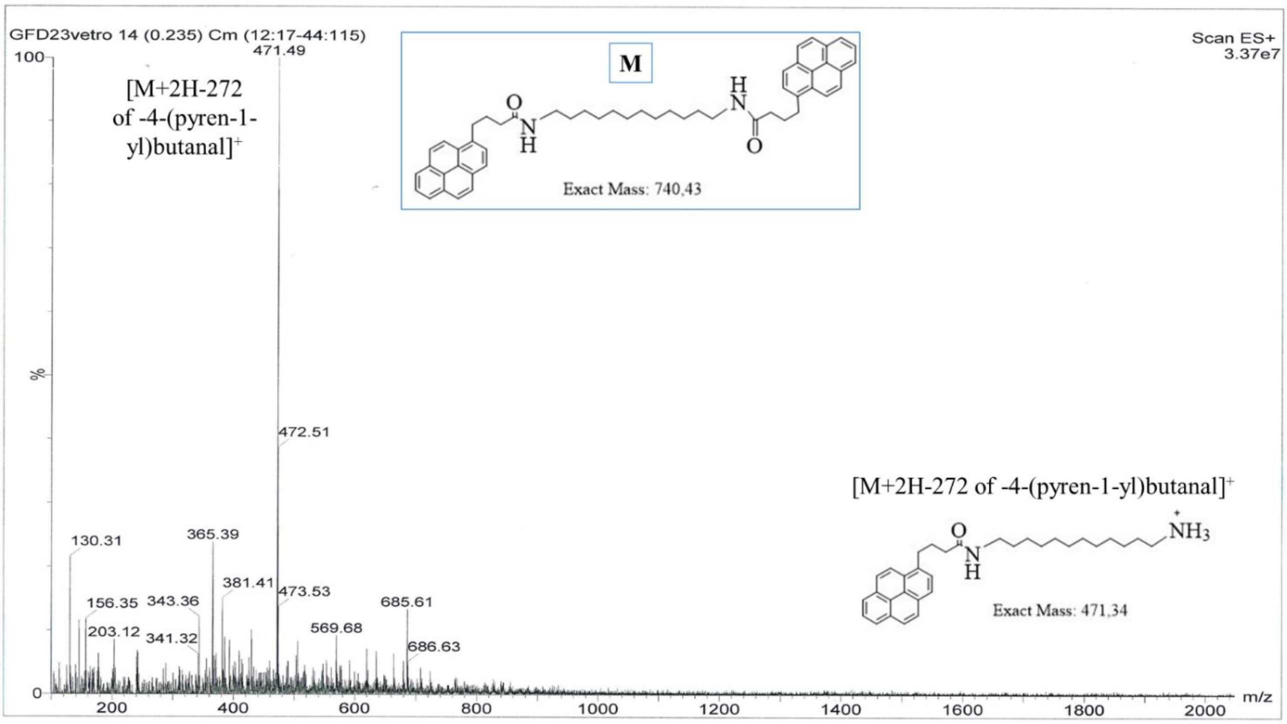

*Figure S15: ESI$^+$ MS spectrum of **2e***

S1.6. Comments on UV-Vis spectra

The UV absorbance spectra (Figure S16a) of bispyrene molecules 2a – 2e, exhibit the distinctive absorption bands assigned to pyrene units at 314 nm, 328 nm and 345 nm. Position and relative intensities of these bands are insensitive to the linker chain length between pyrene ends, which is expected because these depend exclusively on π-π* electronic transition of conjugation system of pyrene. On the other hand, there is a wide bathochromic shift of BP spectra, in comparison to pyrene (Figure S16b), related to the higher molecular weight, which changes the dipole moment of chromophore group.

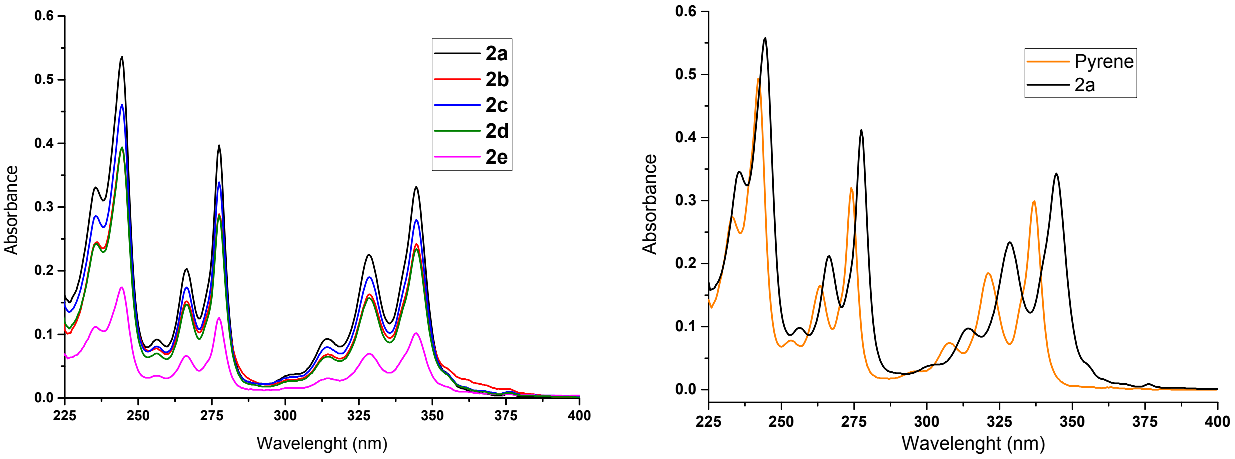

(a) (b)

Figure S16: a) Comparison of UV spectra of solutions (5□10^{-5} M) bispyrene in dichloromethane. b) Comparison between UV-Vis spectrum of solutions of pyrene and BP 2a

S2. Optimization of the concentration of BP for the supramolecular functionalization of GnP with N,N'-(ethane-1,2-diyl)bis(4-(pyren-1-yl)butanamide) (2a)

Based on literature reports [1-3], it was first estimated that the molar concentration to be used should be less than 10^{-3} M to avoid strong BP aggregation[4]. As self-aggregation of BP may compete with GnP interaction, effect of concentration on the interaction with GnP was explored, taking 2a as a model. Solutions of 2a in N,N-dimethylformamide were prepared at concentrations of 10^{-4} M, 10^{-5} M and 10^{-6} M and 0.5 mg mL^{-1} and GnP powder was added in each solution and sonicated to obtain a suspension following the procedure described in the materials and methods section. While the suspensions are left to decant (Figure S17a), part of GnP is precipitated at the bottom of the becker and there is an intresting trend related to 2a concentration in which the highest 2a concentration (10^{-4} M) produces the least stable dispersion, with most of the GnP flakes precipitated after 60 min. On the contrary, decreasing 2a concentration, an increasing concentration of flakes remains in suspension as reflected in the darker coloration. This suggested that a high concentration of 2a results in a cross-linking action, creating a network which readily precipitate, whereas lower concentration provides a less pronounced effect.

Another interesting phenomenon is the light emission observed when the dispersions are lighted at 254 nm. Indeed, as show in the Figure S17 b, while the 2a solution (into the vial) in DMF does not emit, the GnP 2a dispersion (10^{-4} M), showed a clearly visible blue light emission. This experimental evidence suggest a decrease in the energy for the electron excitation, corresponding to a bathochromic shift of fluorescence spectrum to the visible range[5]. This fact support for the π-π the stacking interaction between 2a and π-orbitals on GnP surface, known as Forster energy transfer[6], in which GnP surface adsorbes the light and emit at different wavelenght where the molecule absorbs, shifting to the excited state and emitting in the visible upon relaxation.

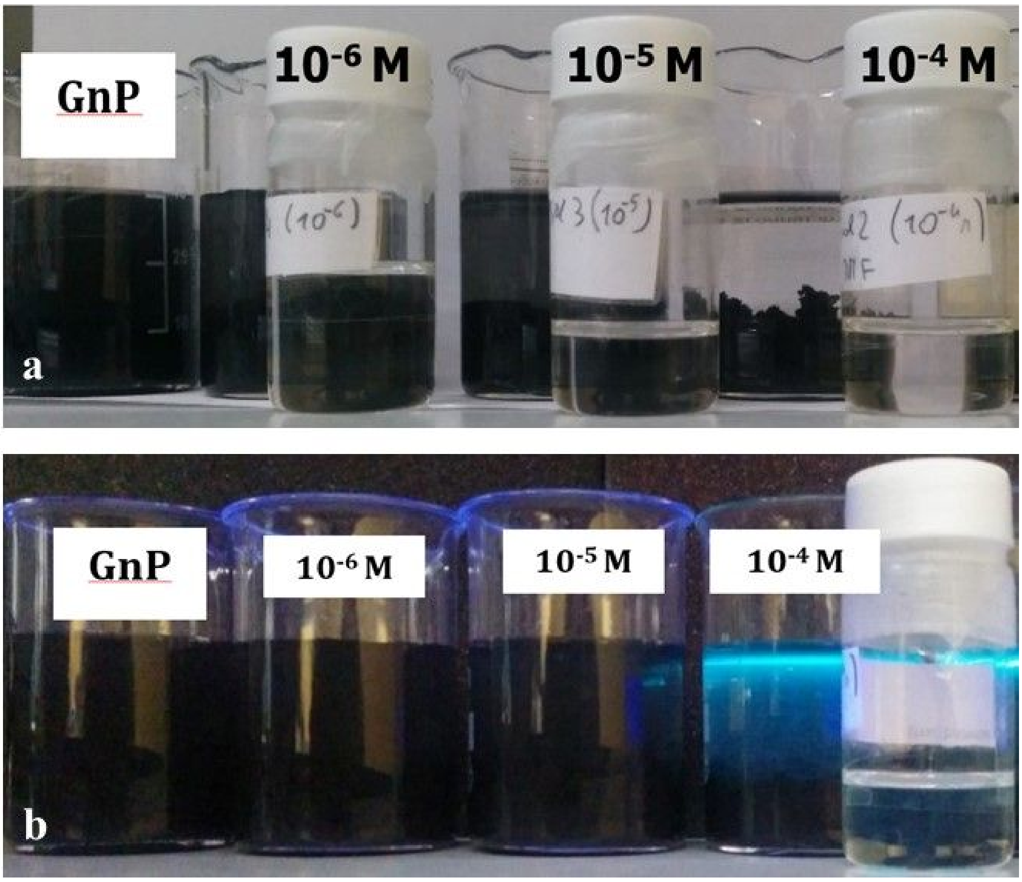

Figure S17. a) In the foreground, into the vials, solutions of 2a in DMF at different concentrations are shown and the respective dispersions with GnP are shown in the background. Inside the beakers, the suspensions were left to decant for one hour and it can be observed that, as the molar concentration of 2a increases, the quantity of GnP precipitated on the bottom increases. b) The solutions and suspensions were lightened at a wavelength of 254 nm and it can be seen that the solution at concentration 10^{-4} does not emit in the visible contrary to its suspension, which instead emits blue light.

The supernatant obtained from each dispersion after centrifugation (30 min, 4000 rpm) was collected and analysed by UV-Vis and fluorescence spectroscopy. For comparison purposes, GnP was also sonicated in DMF, in the absence of bispyrene molecules, and supernatant analysed in the same conditions. From the absorbance spectra (Figure S18), a rough estimation of the concentration of suspended nanoflakes can be obtained by analysing the light absorption at 670 nm (inset Figure S18). This wavelength was chosen because bispyrene do not show any absorption at this wavelength (Figure S19), so the absorbance measured is related exclusively to the concentration of GnP in the dispersions.

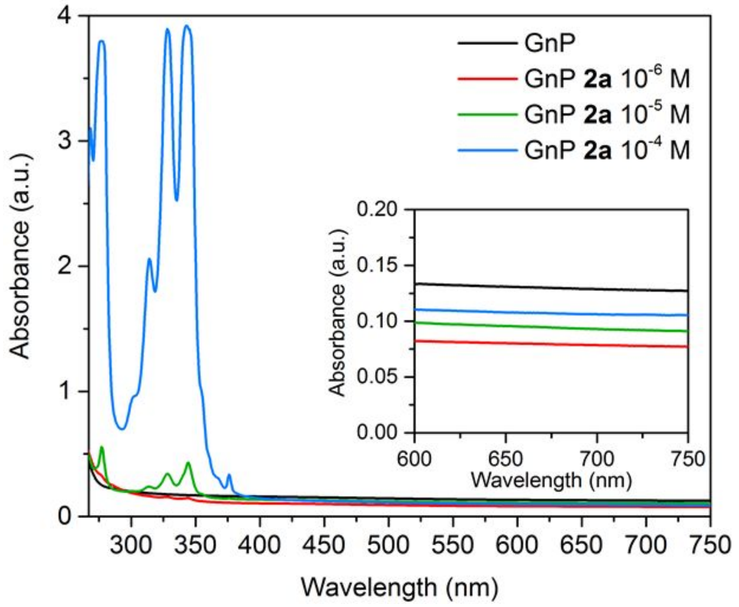

Figure S18. Absorption spectra of GnP and GnP 2a at different concentrations of 2a in DMF. Inset shows a magnification of the 600-750 region

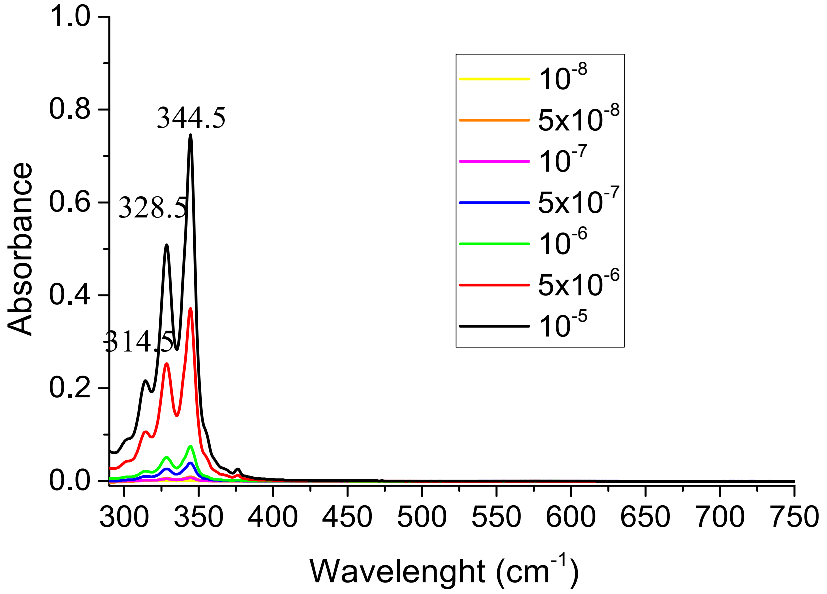

Figure S19. 2a solution absorbance in DMF at different concentration

It can be observed that the concentration of GnP in the suspensions are quite similar and correspond to relatively low absorbance values, suggesting limited concentrations of thin graphene flakes. This is indeed expected, taking into account that the mild sonication condition used were not aimed to obtain an extensive GnP exfoliation, but only to provide sufficient dispersion of GnP flakes, in order to investigate the interactions between 2a and GnP. On the other hand, the well-defined bands of the bispyrene molecule (main absorption at 344, 328 and 314 nm) on the concentrated suspensions GnP 2a 10^{-4} M and GnP 2a 10^{-5} M are clearly observed (Figure S18), indicating that these suspensions

contain a large excess of free 2a molecules. At the lowest concentration, absorption bands for 2a are barely visible. While this is expectable, based on the low concentration, it should also be mentioned that molecules absorbed on GnP are not expected to absorb in the same range, thus further contributing to the low intensity in the UV-VIS spectra.

In order to further investigate the interaction between 2a and GnP, fluorescence measurements were carried out on the same supernatant suspensions. The comparison between fluorescence emission spectra of the GnP 2a dispersions (Figure S20a), compared to solutions of 2a at the different concentrations (Figure S20a, inset) may indeed provide further insight on the organization of bispyrene molecules. Spectra for 2a in DMF solution are characterized by three well defined emissions peaks at 376 nm, 396 nm and 418 nm, related to a monomer emission from the locally excited pyrene moieties, plus a wide band centred at 485 nm related to the stacking of pyrene moieties [3]. No spectral shifts were observed for 2a in the GnP 2a dispersions at any concentration. However, the emission intensities decrease in the GnP dispersions prepared with initial concentrations of the molecule of 10^{-6} M and 10^{-5} M, likely due to the quenching effect of GnP[7]. Furthermore, the 2a excimer emission band at 485 nm is strongly reduced in the dispersions, as observed by the decrease of the ratio between the intensities of the excimer and monomer emission (I_E/I_M) in Figure S20b. This change mainly depends on the lower concentration of 2a aggregates in the dispersions because part of 2a is adsorbed on the basal planes of GnP[8].

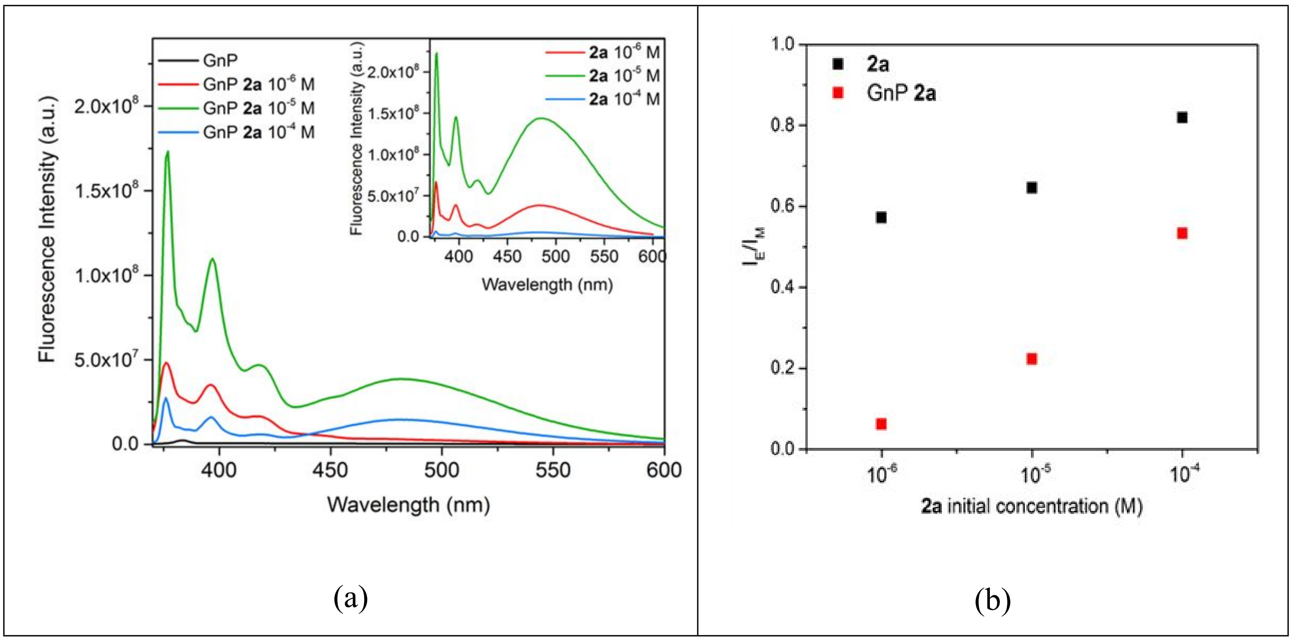

Figure S20. a) Fluorescence spectra (λ_{ex} = 345 nm) of GnP and GnP 2a at different concentrations of 2a in DMF. Inset shows the fluorescence spectra of 2a at the initial concentrations in DMF. b) I_E/I_M ratio for 2a molecule in DMF and for GnP 2a dispersions.

To investigate the organization of 2a onto GnP in the dry state, Raman spectroscopy was carried out on GnP 2a dry powders, obtained by filtration of the suspensions (as reported in materials and methods section). Raman spectra of GnP exhibited a relatively simple structure characterized by two main bands known as the G and D bands. The G band is a sharp band that appears around 1581 cm^{-1} and is related to an in-plane vibrational mode that involve the sp^2 hybridized carbon atoms of the graphite sheet. The D band at 1350 cm^{-1} is often referred to as the defect band or the disorder band, representing a ring breathing mode from sp^2 carbon that becomes active once chemical or structural defects are present on the graphite structure. The analysis of the Raman spectra of the GnP and GnP 2a powders are reported in Figure S21. An increase of the I_D/I_G ratio is observed with increasing the initial concentration of 2a in the suspensions. In particular, a relatively large increase in I_D/I_G ratio is obtained at the highest initial concentration (10^{-4} M), suggesting a high extent of functionalization and possibly an excess of 2a in the form of large aggregates. On the other hand, limited or no increase in the I_D/I_G ratio are observed at lower concentrations, which is compatible with a supramolecular functionalization[9], that is expected to provide only a minor changes in the vibrational properties of graphene layers in GnP.

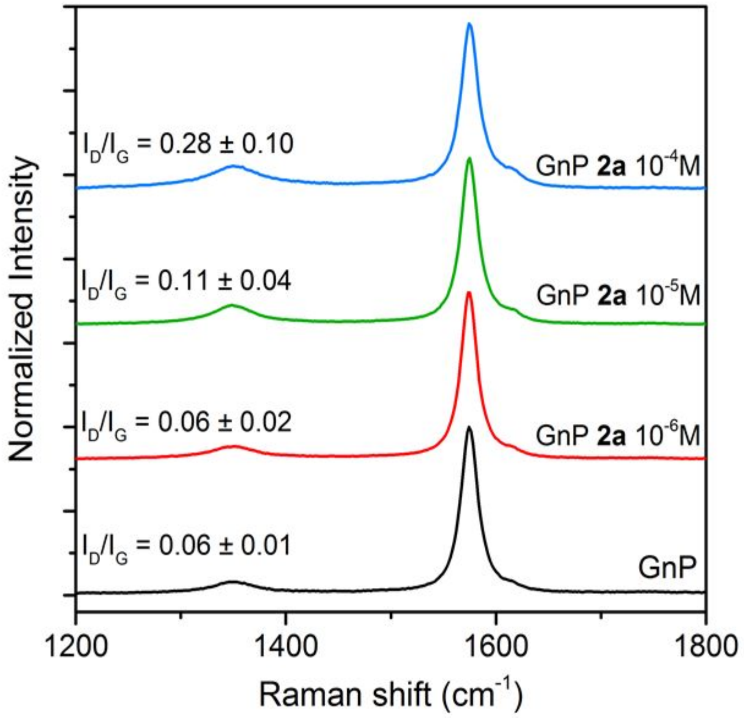

Figure S21.Comparison between Raman Spectra of GnP and GnP 2a powders, normalized on G peak intensity

To complement the analyses of the supernatants and to quantify the amount of 2a molecules effectively adsorbed onto the whole GnP, the suspensions were filtered and the filtrate solution were

analysed by UV-Vis in order to calculate the concentration of 2a in the filtered solution. This was used to indirectly calculate the fraction of 2a adsorbed onto GnP, given the known initial concentration in the GnP 2a suspensions. The fraction of adsorbed bispyrene is 80 % and 55 % of the initial concentration for GnP 2a 10^{-6} M and GnP 2a 10^{-5} M, while it decreases to 10 % in the case of GnP 2a 10^{-4} M, reflecting the large excess of 2a at the highest concentrations (Table S1). Mass fraction of 2a onto the GnP 2a dry powders are also dependent on the initial 2a concentration: a mass fraction as high as 11% was obtained when starting from a 10^{-4} M 2a concentration in the GnP suspension, while significantly lower 2a contents were obtained at lower initial concentrations.

**2a concentration in GnP suspension [M]**	**2a concentration in filtered solution [M]**	**Fraction of 2a adsorbed on GnP [%]**	**Mass fraction of 2a in dry GnP 2a [%]**
**10^{-4}**	0.91 □ 10^{-4}	9	11
**10^{-5}**	0.45 □ 10^{-5}	55	0.71
**10^{-6}**	0.22 □ 10^{-6}	78	0.09

Table S1.Calculation of the mass fraction and mass of 2a absorbed on GnP.

S3. FESEM analysis

FESEM analysis of nanopapers were made to evaluate not only the surface topography but most importantly to understand the cross-sectional organization of the GnP in the presence or BP. Initially, morphology of the nanopapers obtained from GnP suspensions with different concentrations (10^{-4}, 10^{-5} and 10^{-6} M) of 2a were evaluated. Figure S22 shows the comparison between nanopapers fabricated with pristine GnP as well as with those where the GnP were functionalized with different concentration of 2a. The surface topography of pristine GnP nanopaper (Figure S22a) shows the stacking of wrinkled nanoflakes, consistent with the separation and partial fragmentation from the original worm-like structure of this GnP grade, reported elsewhere[10-11]. On GnP 2a prepared with a concentration of 10^{-6} M (Figure S22b), extended light-grey areas are visible, which may be related to the coverage by the BP functionalization. The GnP 2a 10^{-5} M nanopaper (Figure S22c) shows the presence of nanometric clusters onto the GnP flakes, explained by the aggregation of BP. This

phenomenon is apparent in the 10^{-4} M nanopaper, where 2a leads to aggregate whose micrometric dimension are comparable to the dimension of graphene flakes, as clearly observable in Figure S22d.

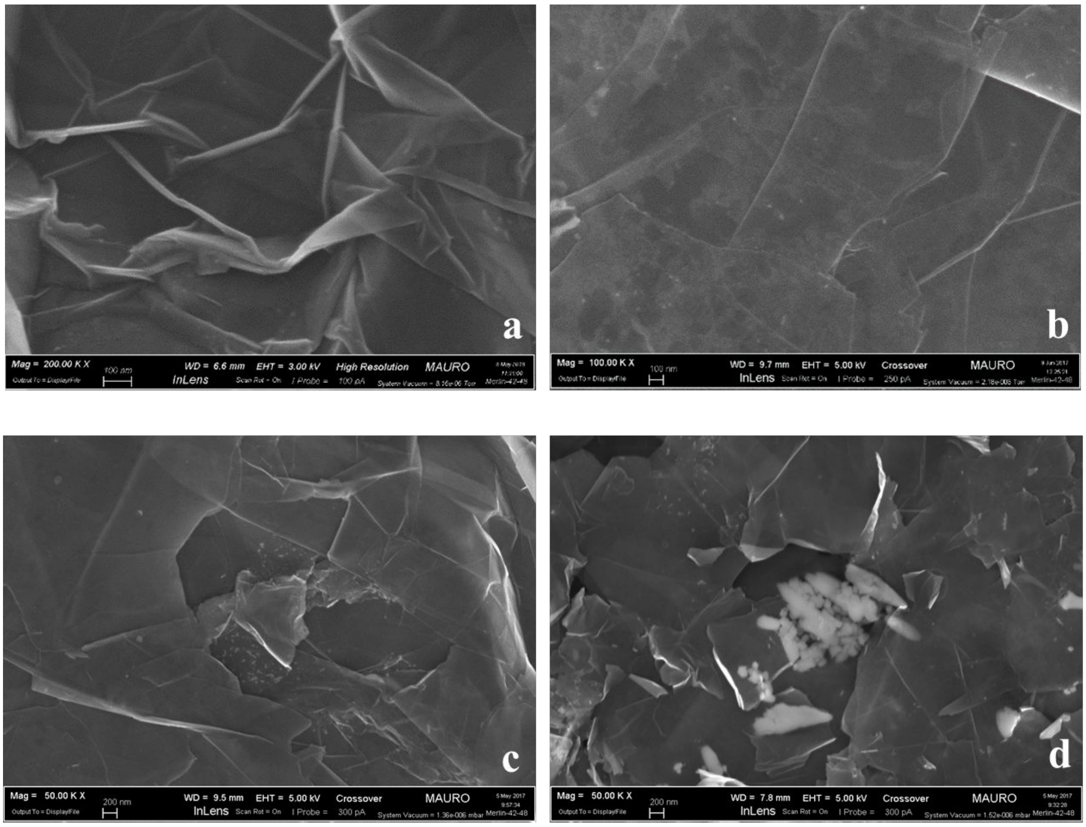

Figure S22. FESEM topography analysis of nanopaper fabricated with pristine GnP (a) and different molar concentration of 2a: 10^{-6} M (b), 10^{-5} M (c) and 10^{-4} M (d).

Based on these evidences, the 10^{-6} M concentration was chosen for the preparation of the nanopapers with all bispyrene derivate molecules (2a-2e) and FESEM analyses were routinely carried out to evaluate the morphological characteristics of the nanopapers. The comparison of surface topographies (Figure S23) shows functionalized nanopapers are less homogeneous compared to pristine GnP, thus confirming the presence of organic functionalization.

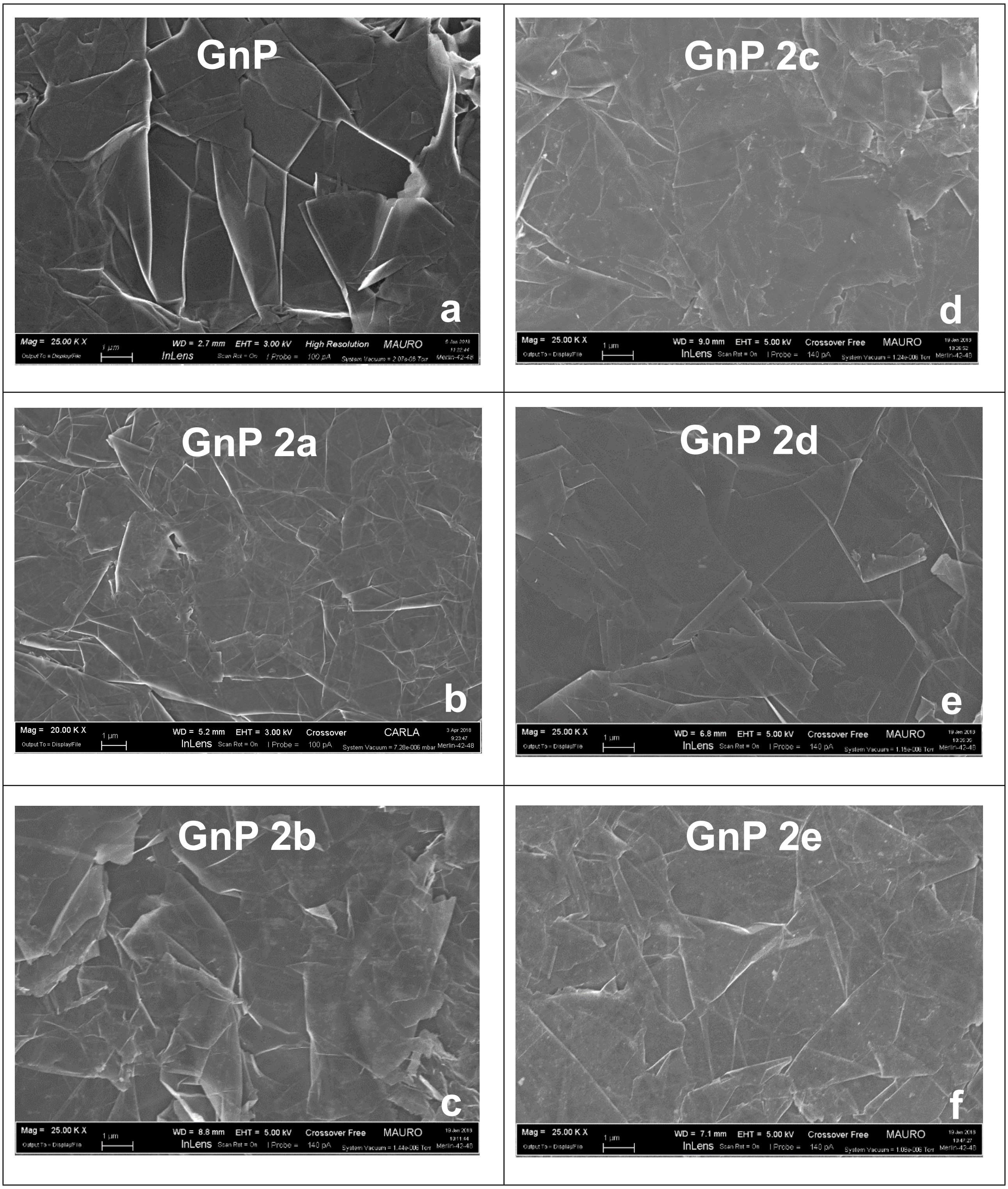

Figure S23. Surface topographies of nanopapers manufactured with GnP (a) and GnP BP (b-f).

Nanopapers cross sections were also routinely observed by FESEM (Figure S24), showing qualitatively similar alignments of the GnP flakes and porosity. Moreover, cross section examinations were used to measure thickness, used to calculate density of each sample (Figure S24).

Densities of nanopapers were found to be significantly different form the reference GnP nanopaper and in particular, lower densities were obtained for all of the GnP-bispyrene nanopapers, ranging between 0.61 g/cm^3 (GnP 2a) and 0.94 g/cm^3(GnP 2e). Interestingly, density values continuously increase with increasing the length of the alkyl chain in BP in GnP-bispyrene nanopaper.

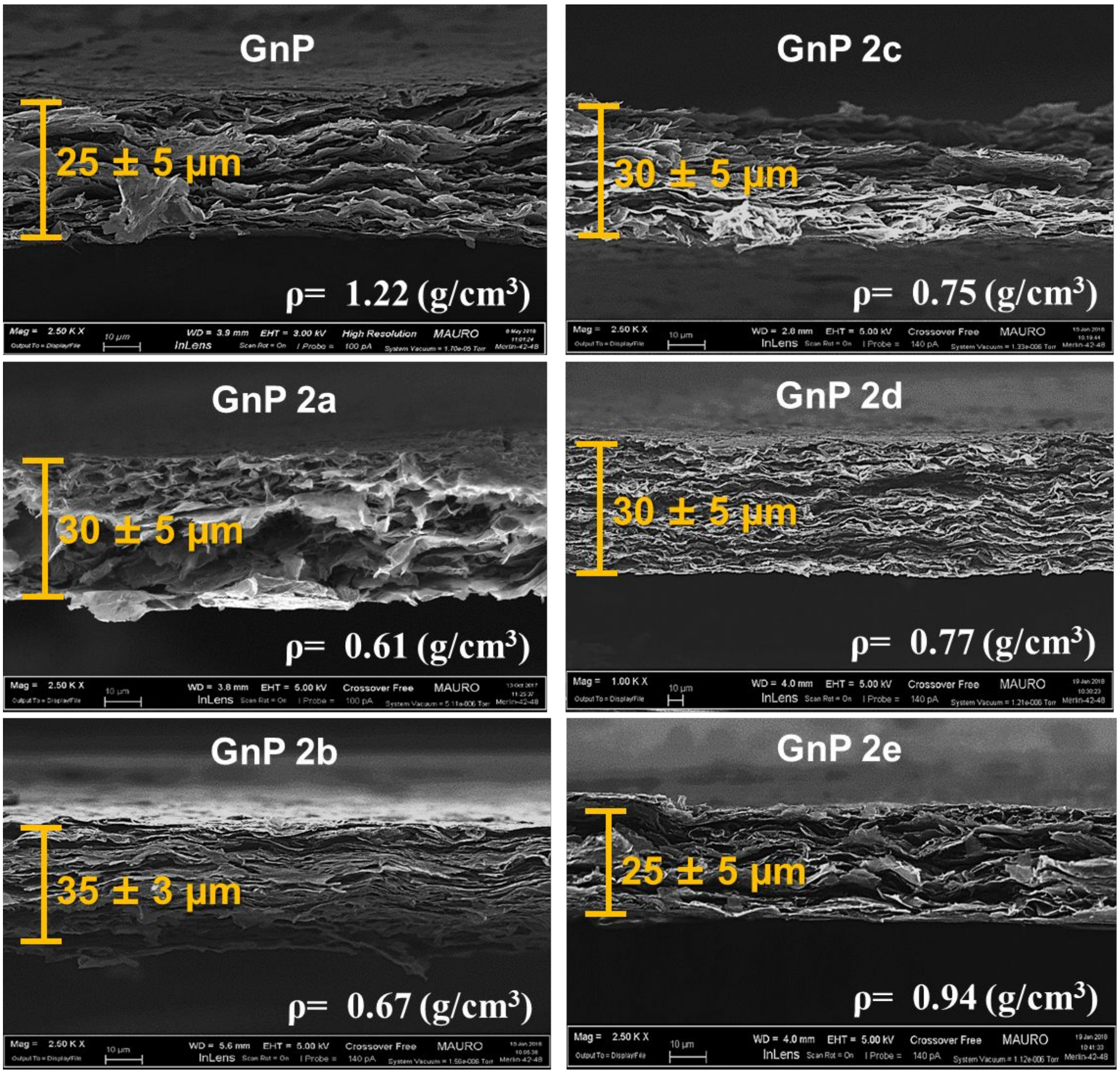

Figure S24. Cross Section FESEM analysis of GnP and GnP BP nanopapers.

S4. Nanoflakes orientation via X-ray diffraction

For each of the GnP BP nanopapers, the experimental curves (one for each angle) were plotted and the peaks were fitted with a Lorentzian function using Origin software, to obtain a representative peak height. These peak values vs. tilt angle were interpolated by an exponential decay function, subsequently integrated over all the 0-90 tilt angle span. The integral value was used to normalize the intensity vs. tilt angle plot, thus allowing comparison between samples, in terms of probability distribution for fractions of flakes oriented from parallel (0° tilt angle) to perpendicular direction, respect to the nanopaper plane. Plots for GnP 2a nanopapers are reported in Figure S25, as an example. Similar plots were obtained for all other nanopapers.

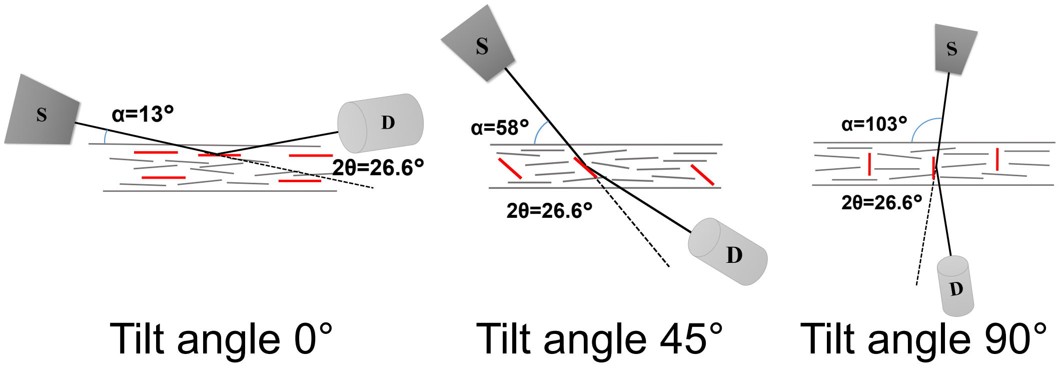

(a)

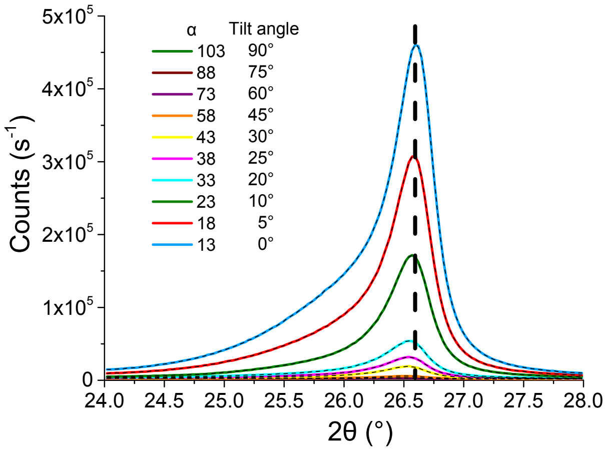

(b)

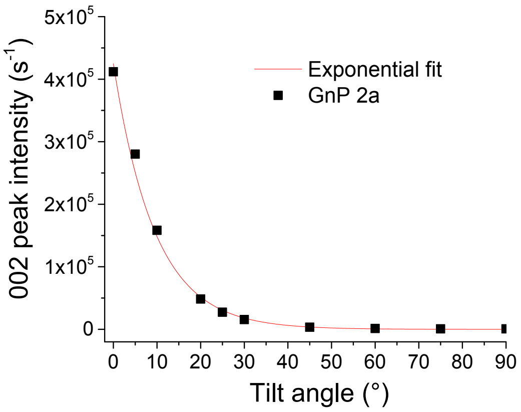

(c)

Figure S25. a) Schematic representation of X-ray diffraction configuration used, as a function of indicident angle, while maintaining constant 2θ=26.6°. In red are highlighted GnP flakes contributing to the diffracted beam, oriented at 0° 45° and 90° tilt angles, respectively. S is for X-ray source, D is for X-ray detector. b) Experimental curves (black dashed plots) and Lorenzian fittings (colour solid plots) for GnP 2a nanopapers, obtained varying the tilt angle from which detect the signal 002 of graphite (2θ=26.6). c) Exponential fitting for Intensity vs Tilt Angle, for GnP 2a nanopapers.

S5. Thermal Conductivity of GnP nanopapers

Thermal conductivity can be calculated from the diffusivity measurement as $k= \alpha \square \rho \square C_p$, where ρ and C_p are respectively the density and the thermal capacity of the material. Experimentally measured densities and diffusivity values for the different nanopapers are reported in Table S2, along with thermal conductivity values, assuming C_p=0.71 $J(gK)^{-1}$ as for graphite. The pristine GnP nanopaper shows the highest in-plane thermal conductivity (151 $Wm^{-1}K^{-1}$) while lower values are obtained for nanopapers containing BP (90-115 $Wm^{-1}K^{-1}$), mainly reflecting their significantly lower density. On the other hand, cross-plane thermal conductivities for GnP BP nanopapers typicslly show higher values than pristine GnP (0.4 $Wm^{-1}K^{-1}$). In fact, for the shorter BP (2a – 2b) cross-plane thermal conductivity are respectively 1.0 $Wm^{-1}K^{-1}$ and 1.1 $Wm^{-1}K^{-1}$, while as the length of the molecule increases, decreasing values are obtained with longer BP, down to 0.4 $Wm^{-1}K^{-1}$ for the nanopaper GnP 2e.

Nanopapers were also compared with copper foil (30 μm), which is the most widely used metal for thermal management applications. However, a fair comparison between the different heat spreader materials cannot be done without taking into account the porosity of GnP nanopapers, directly affecting density and thus reducing thermal conductivity.

	**ρ (gcm⁻³)**	**α in-plane (mm²s⁻¹)**	**α cross-plane (mm²s⁻¹)**	**k in-plane (Wm⁻¹K⁻¹)**	**k cross-plane (Wm⁻¹K⁻¹)**
**GnP**	1.22 ± 0.05	175 ± 11	0.4 ± 0.1	151 ± 12	0.4 ± 0.1
**GnP 2a**	0.62 ± 0.02	204 ± 10	2.2 ± 0.2	90 ± 5	1.0 ± 0.1
**GnP 2b**	0.67 ± 0.02	192 ± 10	2.3 ± 0.2	92 ± 5	1.1 ± 0.1
**GnP 2c**	0.75 ± 0.02	168 ± 13	1.5 ± 0.3	90 ± 7	0.8 ± 0.2
**GnP 2d**	0.77 ± 0.02	167 ± 15	1.3 ± 0.7	92 ± 9	0.7 ± 0.4
**GnP 2e**	0.94 ± 0.02	172 ± 10	0.6 ± 0.2	115 ± 7	0.4 ± 0.1
**Cu**	8.9	-	-	~ 400	

Table S2. Thermal proprieties comparison between GnP nanopapers and copper

To discuss the effect of nanopapers density on their thermal conductivity value, a series of pristine GnP nanopapers having different densities (Table S3) were prepared, by varying the uniaxial compressive load and time.

Nanopaper porosity (φ) was calculated as:

$$\varphi = 1 - \frac{\rho_{nanopaper}}{\rho_{GnP}} \qquad \text{(eq. 1)}$$

where $\rho_{nanopaper}$ is the density (calculated as the mass/volume of a die cut disk), ρ_{GnP} is the density of an individual graphite nanoplatelet, assumed equal to bulk graphite, i.e. 2.2 gcm^{-3}.

As expected, thermal diffusivity values obtained on nanopapers with different porosity does not vary significantly and are confirmed in the range of 175 mm^2s^{-1}. On the other hand, thermal conductivity values obtained for the nanopapers were found in the range from 49 $Wm^{-1}K^{-1}$ (for the highest porosity nanopaper) to 219 $Wm^{-1}K^{-1}$ for the most dense nanopaper.

GnP nanopaper	ρ (gcm^{-3})	porosity (φ)	$\alpha_{//}$ (mm^2s^{-1})	$k_{//}$ ($Wm^{-1}K^{-1}$)
**A**	0.39 ± 0.02	0.83 ± 0.01	178 ± 5	49 ± 3
**B**	0.78 ± 0.03	0.64 ± 0.02	178 ± 3	99 ± 4
**C**	1.17± 0.03	0.47 ± 0.02	169 ± 7	141 ± 6
**D**	1.19± 0.03	0.46 ± 0.02	176 ± 7	149 ± 7
**E**	1.27 ± 0.05	0.42 ± 0.03	179 ± 4	163 ± 7
**F**	1.78 ± 0.05	0.19 ± 0.03	173 ± 3	219 ± 7

Table S3. Thermal diffusivities and thermal conductivities values for pristine GnP nanopapers with variable densities

The problem of heat conduction in heterogeneous materials consisting of a solid continuous phase and a filler dispersed phase, dates back to the early works by Maxwell and Rayleigh, in which different models were proposed to predict the effective thermal conductivity of various types of composite materials. Such models may be applied also to porous media, in which the dispersed phase is air. Different mathematical models were previously proposed for the correlation of thermal conductivity and porosity, including Maxwell-Eucken model, the linear model and effective medium

theories (EMT)[12]. Such models were applied to the experimental data to identify the best fitting, aiming at calculating thermal conductivity of the continuous phase, made of the GnP network, this parameter being independent on the nanopaper porosity. In Figure S26, the experimental results (black points) are reported against linear and Maxwell Eucken models, where $k_{nanopaper}$ is the thermal conductivity of the film, $k_{network}$ is the thermal conductivity of GnP network (including interfacial effect between GnP flakes related to functionalization with BP), k_{air} is the thermal conductivity of air (0.026 W□m^{-1}□K^{-1}) φ is the nanopaper porosity.

Linear model:

$$k_{nanopaper} = k_{network}(1-\varphi) + k_{air}\varphi \qquad \text{(eq. 2)}$$

Maxwell Eucken model:

$$k_{nanopaper} = k_{network}\frac{2k_{network} + k_{air} - 2(k_{network} - k_{air})\varphi}{2k_{network} + k_{air} + (k_{network} - k_{air})\varphi} \qquad \text{(eq. 3)}$$

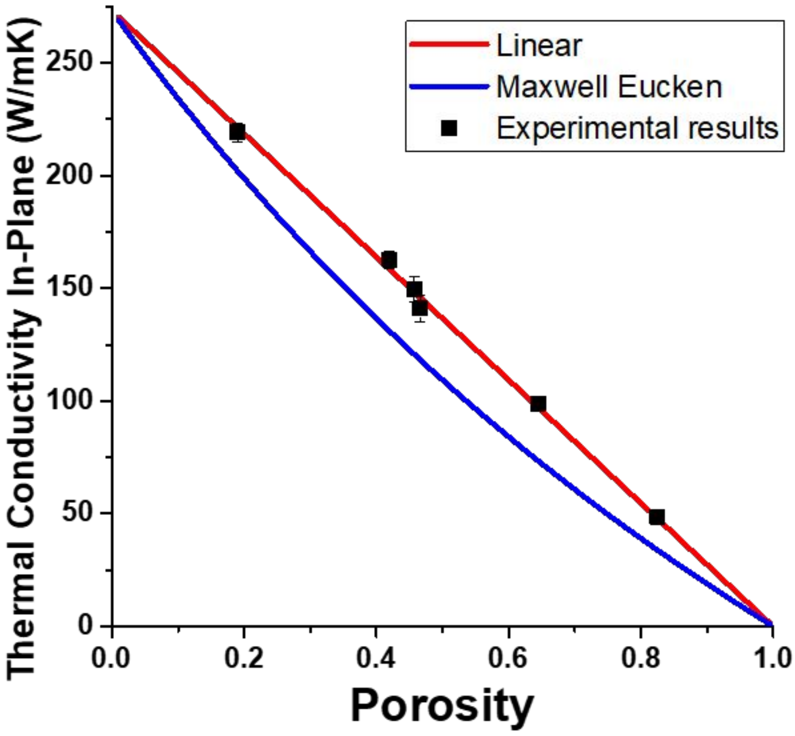

Figure S26: Experimental results for in plane thermal conductivity vs nanopaper porosity, against fits with linear model and Maxwell Eucken model

It is evident that the linear model fit the experimental points better than Maxwell Eucken model, evidencing for a linear dependence of the thermal conductivity with the volume fraction of GnP. Based on this evidence, the nominal thermal conductivity of nanopaper at zero porosity was extrapolated and obtained equal to 273 W□m^{-1}□K^{-1} for pristine GnP.

The linear model was then applied to the calculation of the theoretical zero-porosity thermal conductivities of nanopapers manufactured with functionalized GnP. This allows getting rid of the effect of their difference in densities and obtaining the “intrinsic” thermal conductivity values of GnP networks, theoretically obtainable if nanopapers could be fully densified. These values are reported in Figure S27 and compared to copper.

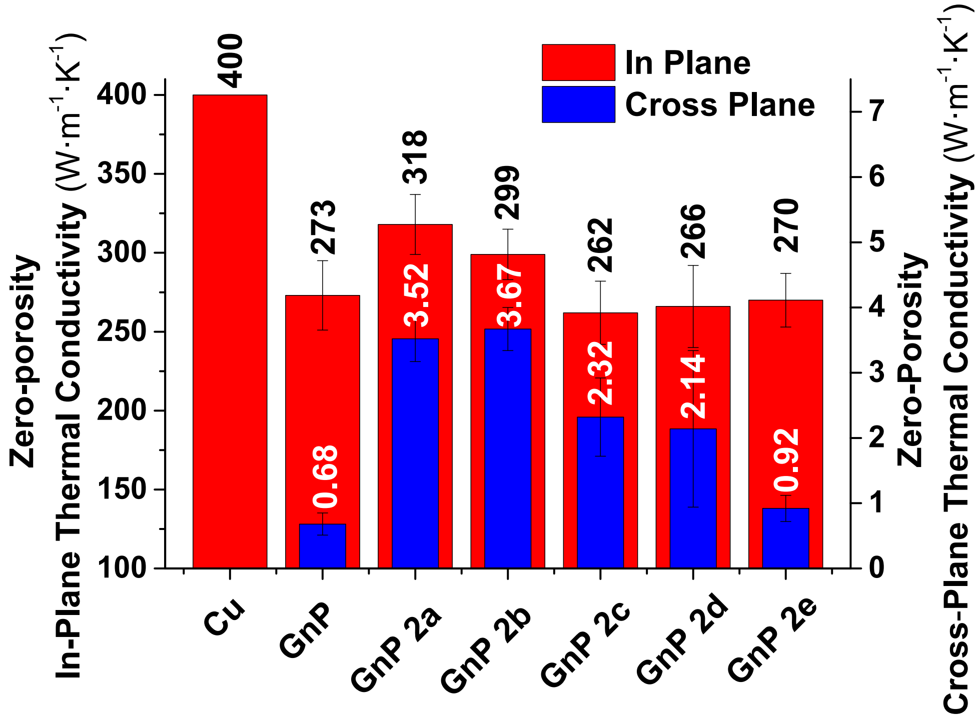

Figure S27. Theroretical zero-porosity thermal conductivity of nanopapers extrapolated by the linear model from porous nanopapers, in comparison with values of copper.

S6. Electrical conductivity

Volumetric electrical conductivity for GnP nanopapers was measured by using the van der Pauw method[13]. The samples were cut into squares of about 1 cm^2, four Ohmic contacts were made at the corners of the nanopapers by means of Ag conductive paste and the resistance for the different pairs of contacts was measured by using a Keithley 2410 sourcemeter. The obtained sheet resistance was multiplied by the thickness of the sample and the GnP volume fraction. Then, the conductivity was calculated by taking the reciprocal of the resistivity thus obtained. The conductivity values are reported in Figure S28, which are in the range of $2 \cdot 10^5$ S/m, with variations among the different nanopapers that are comparable with the experimental error. The uncertainties were calculated by

taking into account variations in the measured resistance values and the uncertainty on the geometrical parameters.

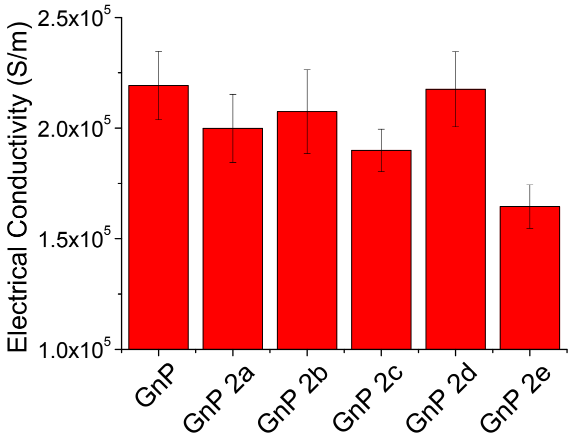

Figure S28: Electrical conductivity for GnP nanopapers

S7. Heat spreader setup and analysis of temperature profiles

The actual setup for the evaluation of heat spread performance of GnP nanopapers, compared to Copper benchmark is shown in Figure S29. Thermal images acquired in time were systematically analysed to extract temperature profiles vs. time and vs. radial coordinate.

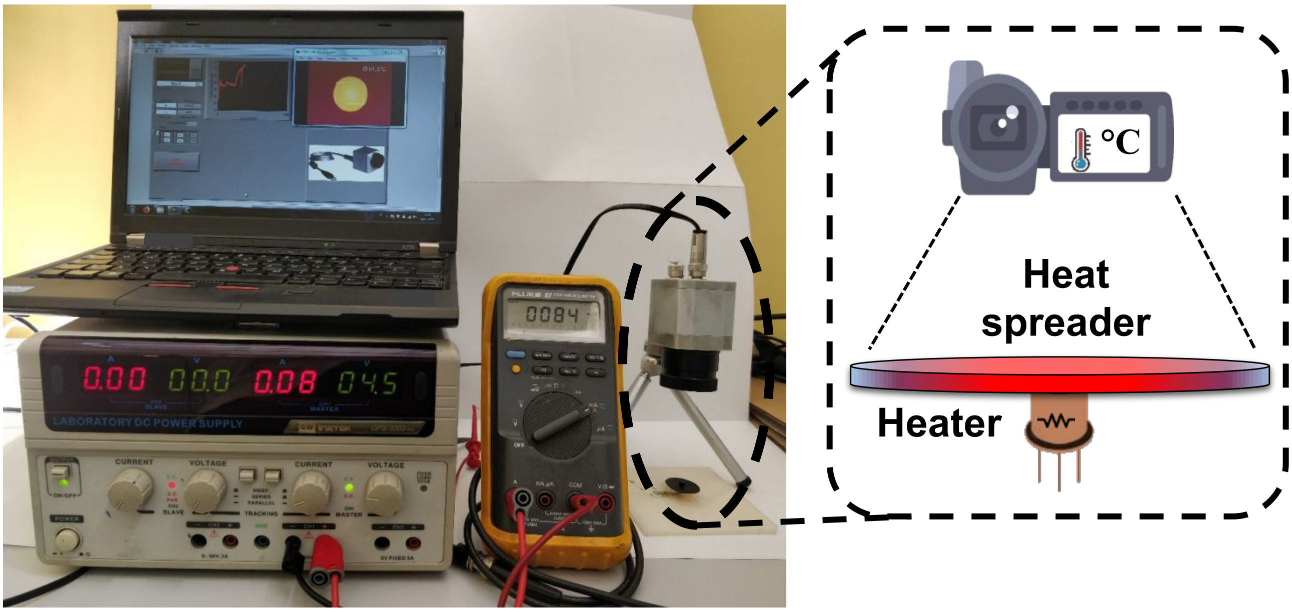

Figure S29: Heat spread test setup and schematic view

Hotspot temperature profiles were recorded on heating (up to 300 s) and on cooling (additional 300s). Temperature plots are reported in Figure S30.

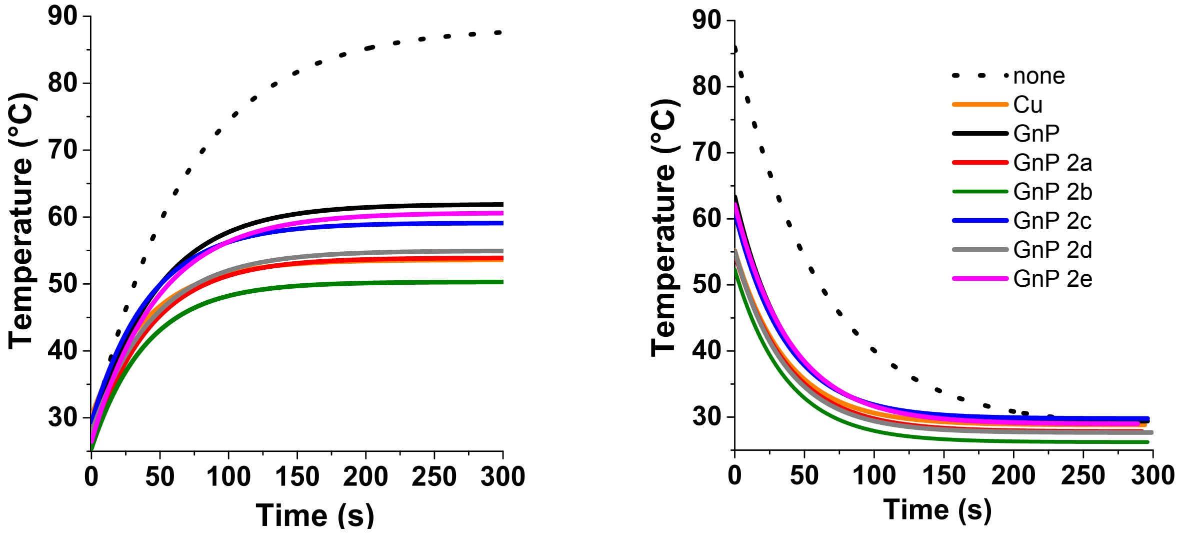

Figure S30: Temperature profiles on heating (left) and cooling (right) for the different heat spreaders tested

Thermal maps for the different heat spreaders, compared with the heater without heat spreader foil, as a function of time on heating (0, 30, 60s) and on cooling (300, 330 and 360s) are reported in Figure S31 and Figure S32, respectively.

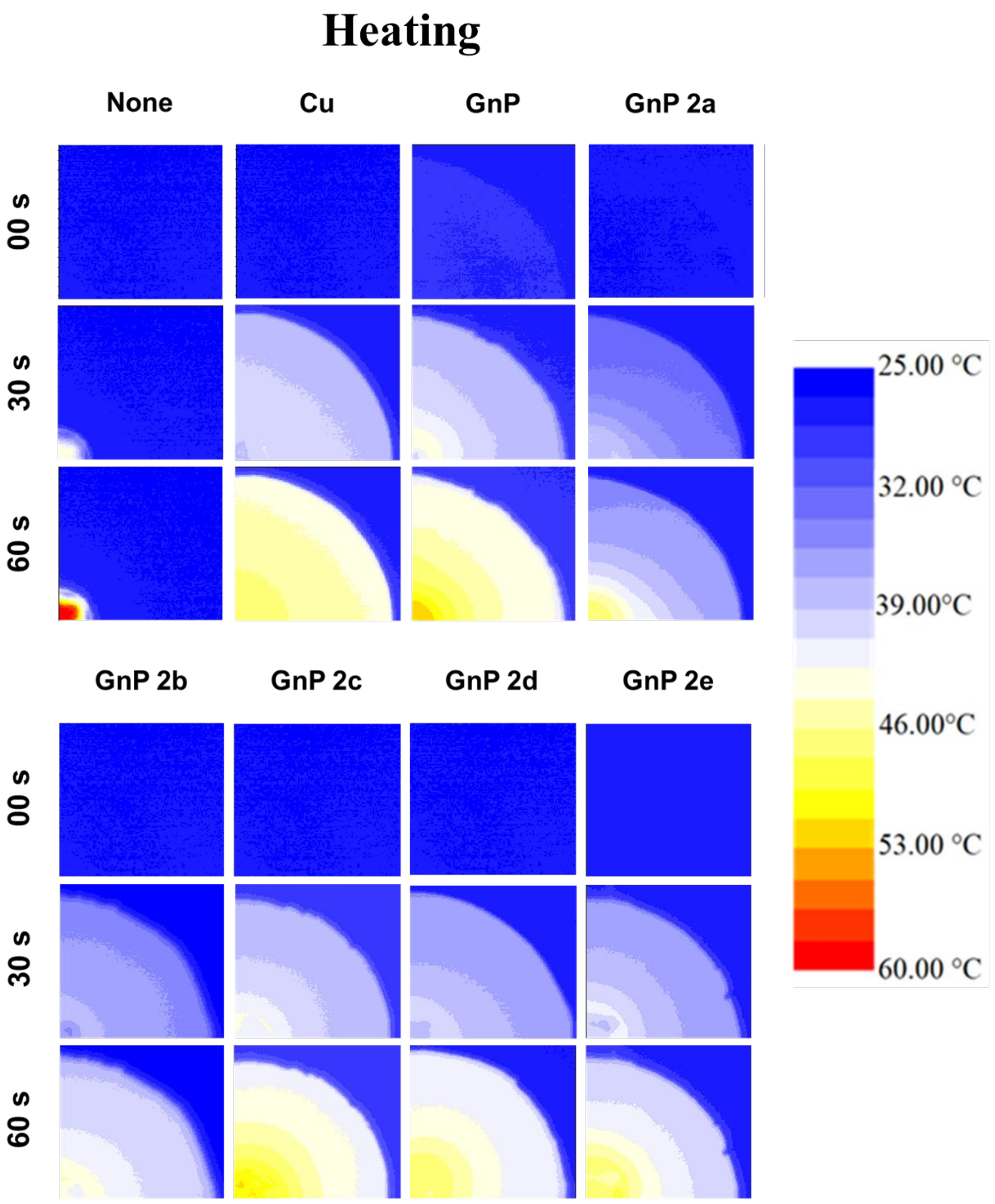

Figure S31: IR thermal images acquired in the initial stages of heating (0, 30 and 60 s) for the different heat spreader foils (I quadrant only for better visibility)

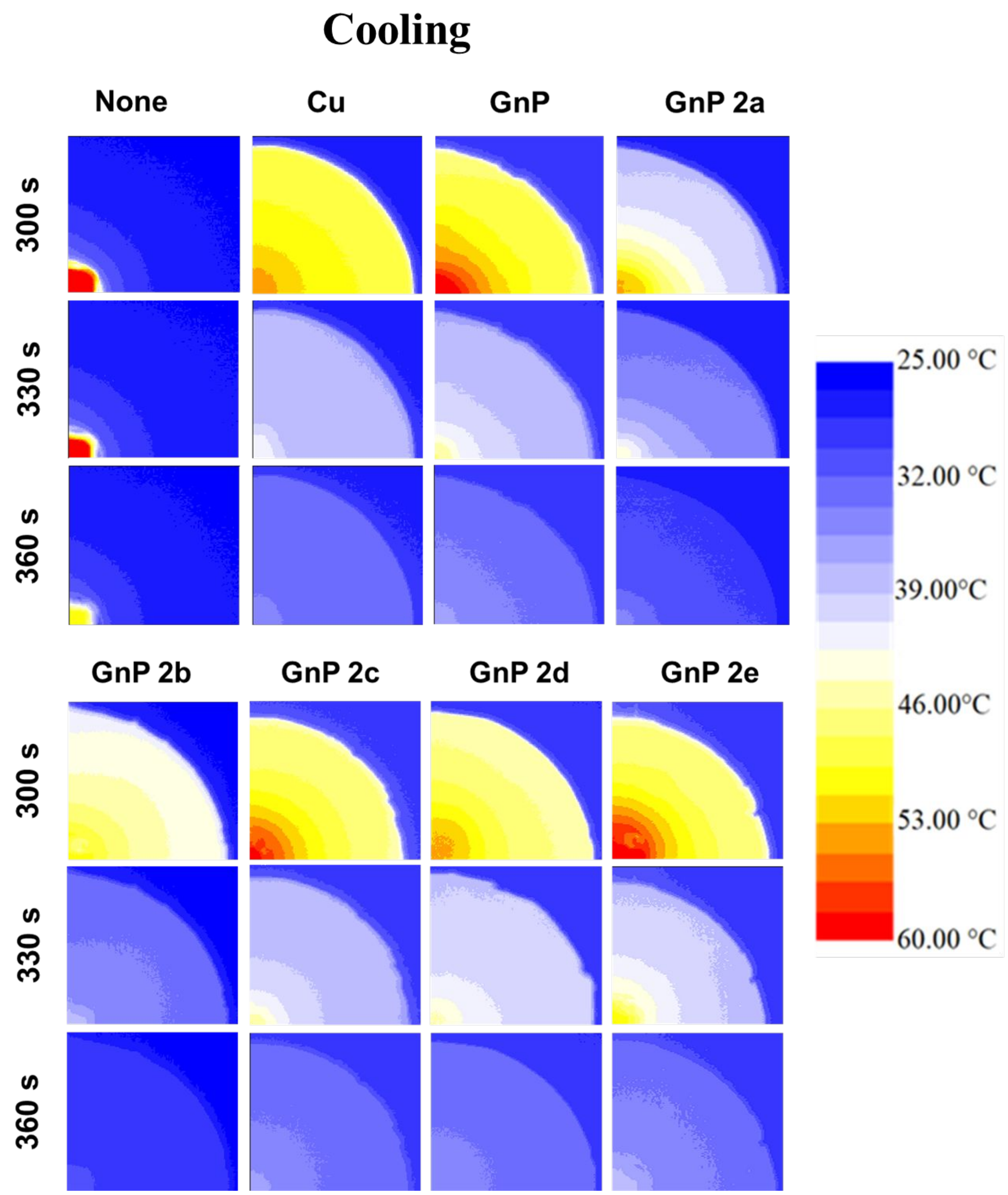

Figure S32. IR thermal images acquired in the initial stages of cooling (300, 330 and 360 s) for the different heat spreader foils

Temperature profiles for each nanopaper were automatically extracted from such thermal maps, along three different directions (x, y axes and bisector) in order to obtain a representative average. Then, the average temperature vs. radius profile was fitted with an exponential decay function, for all the different heat spreader (Figure S33). It is worth noting that decay rates for GnP nanopapers are

significantly higher than for copper foil, reflecting the steepest temperature gradients vs. radius, coherently with the lower thermal conductivity of the nanopapers compared to copper.

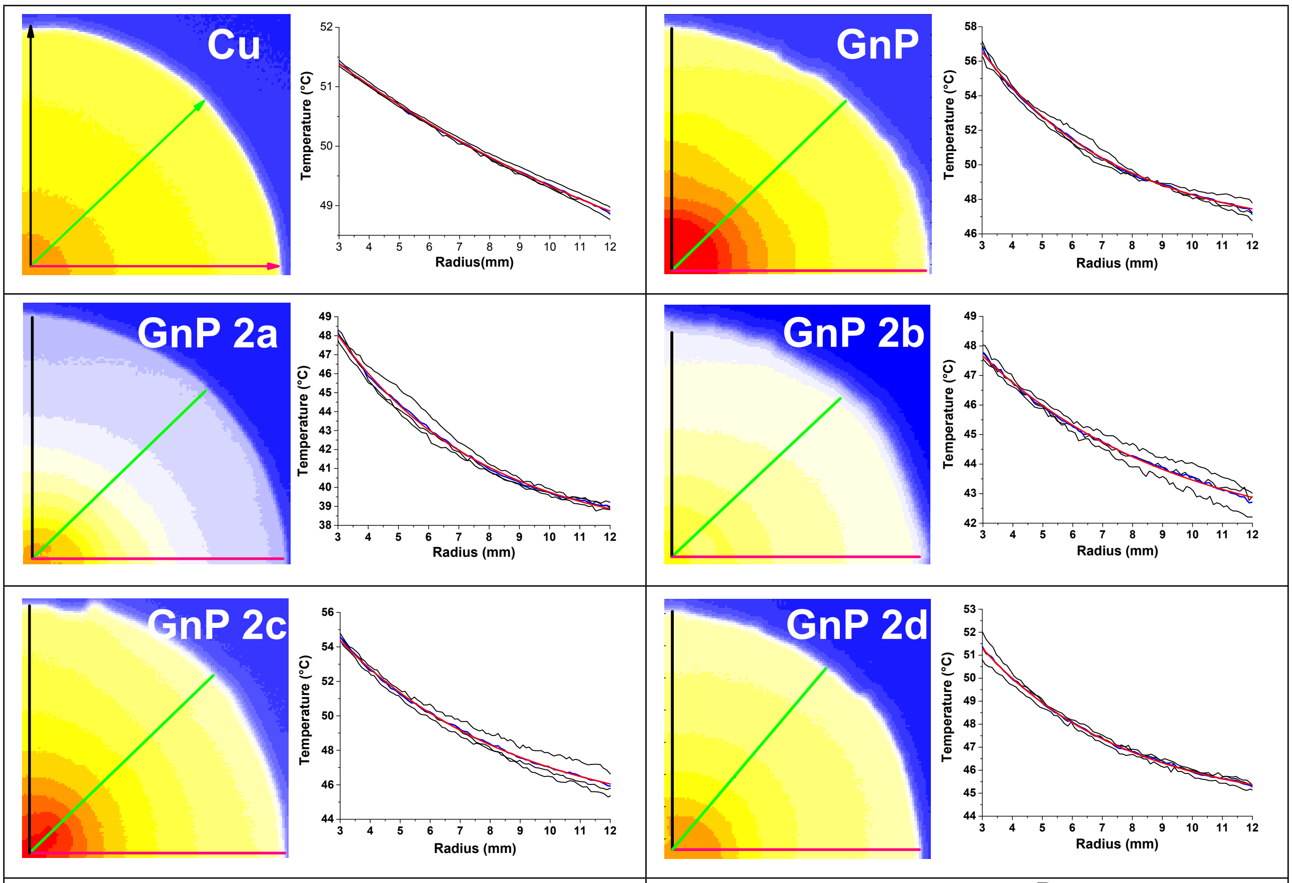

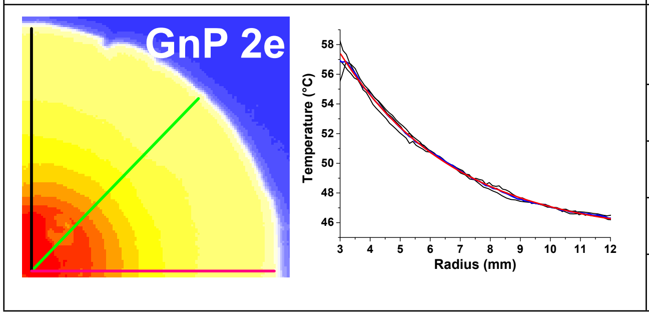

$$T = Ae^{-R_0 x}$$

Cu	R_0= 0.07	GnP 2c	R_0= 0.166
GnP	R_0= 0.217	GnP 2d	R_0= 0.229
GnP 2a	R_0= 0.204	GnP 2e	R_0= 0.259
GnP 2b	R_0= 0.151		

Figure S33: Fitting of temperature vs radius profiles. In black are temperature plots on the different directions (marked on the thermal maps), in blue is the average plot and in red is the exponential fit. Decay rates for fitting plots are also reported

References

1. Cunha, E.; Proença, M. F.; Pereira, M. G.; Fernandes, M. J.; Young, R. J.; Strutyński, K.; Melle-Franco, M.; Gonzalez-Debs, M.; Lopes, P. E.; Paiva, M. D., Water Dispersible Few-Layer Graphene Stabilized by a Novel Pyrene Derivative at Micromolar Concentration. *Nanomaterials* **2018,** *8* (9), 675.

2. Wang, C.; Wang, Z.; Zhang, D.; Zhu, D., Thermal Modulation of the Monomer/Excimer Fluorescence for Bispyrene Molecules through the Gel–Solution Transition of an Organogel: A Thermo-Driven Molecular Fluorescence Switch. *Chemical Physics Letters* **2006,** *428* (1), 130-133.

3. Liu, T.; Huang, Z.; Feng, R.; Ou, Z.; Wang, S.; Yang, L.; Ma, L.-J., An Intermolecular Pyrene Excimer-Based Ratiometric Fluorescent Probes for Extremely Acidic pH and its Applications. *Dyes and Pigments* **2020,** *174*, 108102.

4. Li, W.; Wang, L.; Zhang, J.-P.; Wang, H., Bis-pyrene-based Supramolecular Aggregates with Reversibly Mechanochromic and Vapochromic Responsiveness. *Journal of Materials Chemistry C* **2014,** *2* (10), 1887-1892.

5. Zaharie-Butucel, D.; Potara, M.; Craciun, A. M.; Boukherroub, R.; Szunerits, S.; Astilean, S., Revealing the Structure and Functionality of Graphene Oxide and Reduced Graphene Oxide/Pyrene Carboxylic Acid Interfaces by Correlative Spectral and Imaging Analysis. *Physical Chemistry Chemical Physics* **2017,** *19* (24), 16038-16046.

6. Achadu, O. J.; Managa, M.; Nyokong, T., Fluorescence Behaviour of Supramolecular Hybrids Containing Graphene Quantum Dots and Pyrene-Derivatized Phthalocyanines and Porphyrins. *Journal of Photochemistry and Photobiology A: Chemistry* **2017,** *333*, 174-185.

7. Kim, J.; Cote, L. J.; Kim, F.; Huang, J., Visualizing Graphene Based Sheets by Fluorescence Quenching Microscopy. *Journal of the American Chemical Society* **2010,** *132* (1), 260-267.

8. Huang, Y.; Xing, J.; Gong, Q.; Chen, L.-C.; Liu, G.; Yao, C.; Wang, Z.; Zhang, H.-L.; Chen, Z.; Zhang, Q., Reducing Aggregation Caused Quenching Effect through Co-Assembly of PAH Chromophores and Molecular Barriers. *Nature Communications* **2019,** *10* (1), 169.

9. Wang, J.; Chen, Z.; Chen, B., Adsorption of Polycyclic Aromatic Hydrocarbons by Graphene and Graphene Oxide Nanosheets. *Environmental Science & Technology* **2014,** *48* (9), 4817-4825.

10. Colonna, S.; Bernal, M. M.; Gavoci, G.; Gomez, J.; Novara, C.; Saracco, G.; Fina, A., Effect of Processing Conditions on the Thermal and Electrical Conductivity of Poly (butylene terephthalate) Nanocomposites Prepared via Ring-Opening Polymerization. *Materials & Design* **2017,** *119*, 124-132.

11. Colonna, S.; Battegazzore, D.; Eleuteri, M.; Arrigo, R.; Fina, A., Properties of Graphene-Related Materials Controlling the Thermal Conductivity of Their Polymer Nanocomposites. *Nanomaterials* **2020,** *10* (11), 2167.

12. Gong, L.; Wang, Y.; Cheng, X.; Zhang, R.; Zhang, H., Thermal Conductivity of Highly Porous Mullite Materials. *International Journal of Heat and Mass Transfer* **2013,** *67*, 253-259.

13. Van der Pauw, L. J., A Method of Measuring the Resistivity and Hall Coefficient on Lamellae of Arbitrary Shape. *Philips Technical Review* **1958,** *20*, 220-224.
